# Comparative Efficacy of *Antrodia cinnamomea* on Liver Function Biomarkers in Mice and Rats: A Network Meta-Analysis

**DOI:** 10.3390/antiox14060660

**Published:** 2025-05-30

**Authors:** Chien-Liang Kuo, Berne Ting, Ray Jui-Hung Tseng, Shih-Ping Liu, Jun-Yang Liou

**Affiliations:** 1 Ph.D. Program for Aging, College of Medicine, China Medical University, Taichung 404328, Taiwan; u109048001@cmu.edu.tw (C.-L.K.); berne.ting@uspace.hk.edu.tw (B.T.); 2 School of Medicine, College of Medicine, National Cheng Kung University, Tainan 701401, Taiwan; i54131046@gs.ncku.edu.tw; 3 Center for Translational Medicine, China Medical University Hospital, Taichung 404328, Taiwan; 4 Institute of Cellular and System Medicine, National Health Research Institutes, Zhunan 350401, Taiwan; 5 Graduate Institute of Biomedical Sciences, China Medical University, Taichung 404328, Taiwan

**Keywords:** *Antrodia cinnamomea*, network meta-analysis, liver function, animal models, biomarkers

## Abstract

This study systematically evaluates the hepatoprotective effects of different types and doses of *Antrodia cinnamomea* extracts (triterpenoids, polysaccharides, and ubiquinone derivatives) on liver function biomarkers, including alanine aminotransferase (ALT), aspartate aminotransferase (AST), malondialdehyde (MDA), and tumor necrosis factor-alpha (TNF-α), using a network meta-analysis (NMA) approach. Comprehensive literature searches were conducted in PubMed, Embase, Cochrane CENTRAL, and Web of Science databases to identify eligible animal studies involving standardized mouse and rat models. Interventions were categorized based on extract types and dosage levels (high, medium, low), with controls including negative groups (vehicle-treated) and positive groups (e.g., silymarin, N-acetylcysteine). A random-effects model estimated mean differences (MDs) with 95% confidence intervals (CIs), risk of bias was assessed with the SYRCLE tool, and sensitivity analyses verified robustness. The protocol has been registered in INPLASY (INPLASY202540040). The results indicated that triterpenoids, particularly at high and medium doses, were the most effective in reducing ALT (MD: −42.37, 95% CI: −54.19 to −30.54) and AST (MD: −50.18, 95% CI: −73.31 to −27.05). High-dose polysaccharides also showed notable effects, while other interventions demonstrated variable efficacy. For oxidative stress, high-dose triterpenoids showed the most pronounced reduction in MDA (MD: −19.05, 95% CI: −24.00 to −14.09), followed by medium-dose triterpenoids and all-dose polysaccharides. Regarding inflammation, high- and medium-dose triterpenoids significantly reduced TNF-α levels (high-dose MD: −88.75, 95% CI: −119.68 to −57.82; medium-dose MD: −89.27, 95% CI: −125.51 to −53.02), with overlapping confidence intervals indicating similar efficacy. High- and low-dose polysaccharides also demonstrated moderate anti-inflammatory effects. In conclusion, high-dose triterpenoids showed favorable and consistent effects across multiple biomarkers, which highlights their potential value for future liver-related therapeutic strategies.

## 1. Introduction

Liver diseases, particularly metabolic dysfunction-associated steatotic liver disease (MASLD), formerly known as non-alcoholic fatty liver disease (NAFLD), represent a major global health challenge due to their rising prevalence, association with metabolic disorders, and potential progression to severe conditions such as cirrhosis and hepatocellular carcinoma [1,2]. Despite ongoing advances in pharmacological treatments, there remains a critical need for safe, effective, and complementary interventions with fewer side effects [3]. Natural products have increasingly attracted scientific interest for their hepatoprotective properties, offering potential therapeutic benefits through multiple biological mechanisms [4,5].

*Antrodia cinnamomea* (*A. cinnamomea*), a medicinal mushroom native to Taiwan, is distinct from other medicinal mushrooms such as *Ganoderma lucidum* (reishi), *Cordyceps sinensis* (cordyceps), and *Sanghuangporus sanghuang* due to its unique bioactive compounds (e.g., antcin A, antroquinonol) and its exclusive growth on the heartwood of *Cinnamomum kanehirae*. It has long been used in traditional medicine for treating various ailments, including liver dysfunction [6,7]. *A. cinnamomea* contains a variety of bioactive compounds, including polysaccharides (e.g., β-D-glucans), triterpenoids (e.g., antcin A, antcin B, antcin C, dehydrosulfurenic acid), antroquinonol, and various phenolic compounds (e.g., syringic acid, gallic acid). Each of these compounds exhibits promising pharmacological effects, including antioxidative, anti-inflammatory, and hepatoprotective activities [8,9]. Preclinical studies have suggested that different extracts and dosages of *A. cinnamomea* can effectively ameliorate liver damage, reduce oxidative stress, and attenuate inflammatory responses in animal models [10,11].

However, the comparative efficacy of these bioactive components from *A. cinnamomea* remains unclear, particularly in relation to dosage and specific liver function biomarkers [12]. Traditional pairwise meta-analyses have limitations, such as an inability to simultaneously compare multiple interventions and integrate direct and indirect evidence across various studies [13,14]. Network meta-analysis (NMA) addresses these limitations by enabling simultaneous comparisons of multiple interventions, providing a comprehensive understanding of their relative effectiveness [15].

Therefore, this study aimed to systematically evaluate and compare the hepatoprotective efficacy of different bioactive extracts from *A. cinnamomea* on liver function biomarkers—including alanine aminotransferase (ALT), aspartate aminotransferase (AST), malondialdehyde (MDA), and tumor necrosis factor-alpha (TNF-α)—using an NMA of animal experiments. ALT and AST are widely recognized indicators of liver injury and hepatocellular integrity, while MDA reflects oxidative stress levels, and TNF-α serves as a pro-inflammatory marker associated with hepatic inflammation [16,17,18]. By synthesizing existing evidence from preclinical research, we aim to identify the most effective extract types and optimal dosages of *A. cinnamomea* to provide a robust basis for future clinical translation and therapeutic recommendations.

## 2. Methods

In accordance with the PRISMA extension for Network Meta-Analyses (PRISMA-NMA) [19], this study was carefully designed and conducted to maintain methodological transparency and rigor. The research protocol was registered in advance with the International Platform of Registered Systematic Review and Meta-analysis Protocols (INPLASY), under registration number INPLASY202540040.

### 2.1. Database Searches and Study Identification

A comprehensive literature search was conducted using four electronic databases: PubMed, Embase, the Cochrane Central Register of Controlled Trials (CENTRAL), and Web of Science. The search strategy employed Boolean operators to combine relevant terms and identify eligible studies. Search terms included combinations of keywords related to *Antrodia cinnamomea*, hepatoprotection, non-alcoholic fatty liver disease, triterpenoids, polysaccharides, β-glucan, antrodin, and antroquinonol. In addition to database searches, reference lists of relevant review articles were manually screened to identify additional studies not captured through electronic searches [7,12,20].

### 2.2. Inclusion and Exclusion Criteria

To ensure methodological consistency and relevance, specific criteria were applied to determine study eligibility. Included studies were limited to in vivo animal experiments involving murine models, specifically mice (Mus musculus) and rats (Rattus norvegicus). Only commonly used laboratory strains were accepted—such as C57BL/6, BALB/c, and ICR for mice and Sprague Dawley (SD) and Wistar for rats. Studies using other species, including rabbits, pigs, dogs, and non-human primates, were excluded. In vitro studies, such as cell culture experiments, as well as clinical trials involving human participants and secondary research (e.g., systematic reviews, narrative reviews, and meta-analyses) were also excluded. To be eligible, studies had to evaluate liver function and include a control group—either untreated, vehicle-treated (e.g., DMSO, PBS), or a positive control treated with a known hepatoprotective agent such as Silymarin, N-acetylcysteine (NAC), Metformin, or Resveratrol. The intervention group must have received *Antrodia cinnamomea* or its derived extracts, including but not limited to polysaccharides, triterpenoids, and ubiquinone derivatives. The dosage (mg/kg), route of administration (e.g., oral gavage, intraperitoneal injection), and intervention duration (single or repeated dosing) had to be clearly specified. Studies must have also provided quantifiable outcome data, such as mean ± standard deviation or median with interquartile range. Eligible outcomes included at least one primary or secondary indicator. Primary outcomes were serum levels of liver enzymes, particularly AST and ALT. Secondary outcomes included oxidative stress markers such as MDA and inflammatory cytokines such as TNF-α. Studies were excluded if they co-administered *Antrodia cinnamomea* with other plant extracts or pharmacological agents without clearly distinguishing its independent effects. Additionally, studies that failed to report essential methodological details—such as dosage, extraction method, or route of administration—were not considered for inclusion. The dose classification (low/medium/high) in this study followed the original definitions provided in each included study. Duplicates and clearly irrelevant records (e.g., non-animal studies, reviews, editorials) identified through the initial screening process were removed prior to full-text assessment.

### 2.3. Modeling for Network Meta-Analysis

In this NMA, the modeling approach was carefully designed to ensure interpretability and minimize heterogeneity [21]. To maintain coherence in network geometry, we focused on comparisons between different *Antrodia cinnamomea* extract types and control groups. Specifically, the intervention categories included high, medium, and low doses of triterpenoids, polysaccharides, and antroquinonol to reflect the dose frameworks established by each study according to pharmacological properties, toxicity thresholds, and traditional usage. These were compared against both negative and positive controls, as well as model controls. To preserve consistency across comparisons, studies that combined multiple extract types without isolating their individual effects were excluded. The inclusion of only extract-based interventions versus standardized control conditions ensured that the network reflected a clear structure and reduced the potential for inconsistency across treatment nodes. The classification of interventions into distinct dosage categories was based on consensus discussions between two authors (Kuo and Ting), with reference to reported concentrations and administration protocols in the included studies. Any discrepancies in classification were resolved through consultation with a third reviewer (Liu) to ensure consistency and reproducibility in node definition within the network.

### 2.4. Methodological Quality Appraisal

To evaluate the methodological quality of the included animal studies, we adopted SYRCLE’s risk of bias (RoB) tool developed by Hooijmans et al. This tool is specifically tailored for animal intervention studies and was adapted from the Cochrane Collaboration’s RoB framework to account for the unique characteristics of preclinical research [22]. The SYRCLE RoB tool systematically assesses various domains of potential bias, including selection bias (e.g., sequence generation and allocation concealment), performance bias (e.g., random housing and blinding of caregivers), detection bias (e.g., blinding of outcome assessment), attrition bias (e.g., incomplete outcome data), reporting bias (e.g., selective outcome reporting), and other sources of bias that may influence internal validity. Each domain was rated as low, unclear, or high risk of bias. For example, sequence generation was rated as low risk if randomization was clearly described, unclear if details were missing, and high risk if randomization was absent or inappropriate. The percentage of studies with low, unclear, and high risk in each domain was calculated by dividing the number of studies in each category by the total number of included studies. Each included study was independently evaluated by two reviewers (Ting and Tseng), and any discrepancies were resolved through consensus discussions with a third reviewer (Kuo).

### 2.5. Primary Outcome: Improvement in Liver Function

The primary outcome of this network meta-analysis focused on the hepatoprotective effects of *Antrodia cinnamomea,* as measured by improvements in liver enzyme levels. Specifically, we analyzed serum levels of ALT and AST, which are widely recognized biomarkers of liver injury and hepatic cellular integrity [23,24]. Reductions in these enzymes following intervention were used as indicators of liver function improvement across different extract types and dosage levels of *A. cinnamomea*.

### 2.6. Secondary Outcome: Oxidative Stress and Inflammatory Markers

The secondary outcome involved the evaluation of *Antrodia cinnamomea*’s effects on oxidative stress and inflammation, which are key mechanisms in liver damage progression. MDA, a biomarker of lipid peroxidation, was assessed as an indicator of oxidative stress [25]. Additionally, TNF-α, a pro-inflammatory cytokine, was analyzed to evaluate the compound’s potential anti-inflammatory effects [26]. These markers provided insight into the broader biological mechanisms underlying the hepatoprotective properties of *A. cinnamomea*.

### 2.7. Data Extraction, Management, and Conversion

Two reviewers (Ting and Tseng) independently extracted relevant data from each included study using a standardized data extraction form. Extracted information included animal species and strain, sample size, study design, details of *Antrodia cinnamomea* intervention (e.g., type of extract, dosage, route of administration, intervention duration), control group characteristics, and reported outcomes such as ALT, AST, MDA, and TNF-α levels. Any discrepancies between reviewers were resolved through discussion or consultation with a third reviewer (Kuo).

When necessary, when data were not directly reported in the published articles, attempts were made to contact corresponding authors to obtain missing information. For studies that presented data only in graphical form, numerical values were extracted using Fiji/ImageJ version 2.16.0, an open-source image analysis tool [27,28]. When studies reported continuous outcomes using mean and standard error (SE), we converted SE to standard deviation (SD) using the formula SD = SE × √n, where n is the sample size [29]. All extracted data were managed and converted in accordance with the Cochrane Handbook for Systematic Reviews of Interventions and other relevant methodological guidelines to ensure accuracy, consistency, and transparency throughout the meta-analysis process [15,30,31].

### 2.8. Statistical Analysis

In this NMA, a random-effects model was employed to account for heterogeneity across studies using different types and dosages of *Antrodia cinnamomea* extracts [32]. The statistical analysis was conducted using the frequentist approach in MetaInsight (version 6.3.0, National Institute for Health Research Complex Reviews Support Unit, London, UK), a web-based NMA platform that applies the netmeta package in R for computation [33]. Forest plots and network diagrams were initially generated to visualize all pairwise comparisons among the included intervention and control groups. The main analysis focused on calculating mean differences (MDs) with 95% confidence intervals (CIs) for both primary outcomes (ALT and AST levels) and secondary outcomes (MDA and TNF-α levels), comparing each intervention to control groups [34]. Interventions were ranked according to their relative efficacy based on the pooled results from both direct and indirect comparisons, with rankings presented in tabular form. Inconsistency across the network was assessed using appropriate statistical tools within MetaInsight. Statistical significance was defined as a two-sided *p*-value of less than 0.05.

### 2.9. Sensitivity Analysis

To ensure the robustness and reliability of our findings, sensitivity analyses were conducted using complementary strategies. A leave-one-out approach was applied by sequentially excluding each study to determine whether the omission of any single study would significantly alter the overall results or affect the relative ranking of interventions within the network [30]. In certain comparisons, only two studies were available. The exclusion of one study in these cases left a single source of evidence for that particular comparison, thereby preventing the generation of meta-analytic estimates and forest plots. As a result, such comparisons were excluded from arm-level sensitivity analyses due to structural constraints but were retained in the overall study-level analysis to preserve the integrity and comprehensiveness of the findings [35].

### 2.10. Publication Bias

We assessed the potential presence of publication bias in accordance with the guidelines outlined in the Cochrane Handbook for Systematic Reviews of Interventions [36]. Funnel plots were generated using Comprehensive Meta-Analysis software, version 4 (Biostat, Englewood, NJ, USA), focusing on the comparisons between *Antrodia cinnamomea* extract interventions and the control groups. To further evaluate asymmetry in the funnel plots, we conducted Egger’s regression test, which provided a statistical measure of potential small-study effects and publication bias [15]. A *p*-value less than 0.05 was considered indicative of significant publication bias.

## 3. Results

### 3.1. Identification of Research and Construction of Network Models

**Figure 1:**
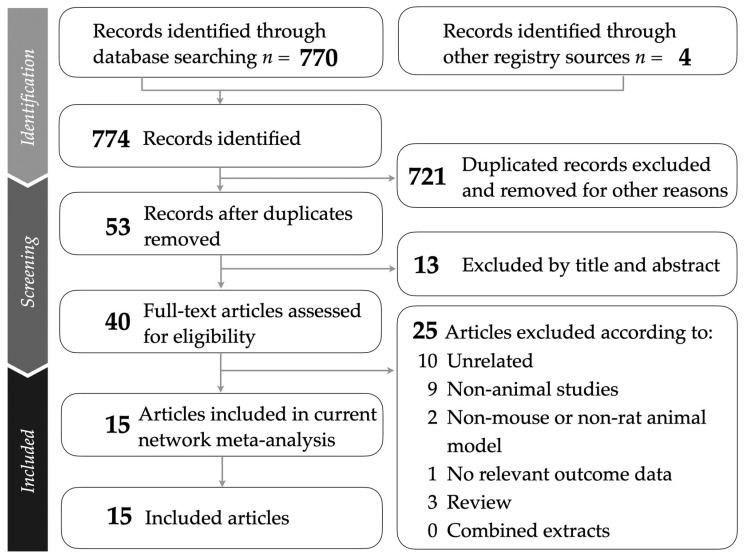
Flowchart presenting the study selection process following PRISMA guidelines.

This study followed the PRISMA guidelines, as illustrated in Figure 1. The PRISMA NMA checklist is provided in Supplementary Table S1 for detailed reference. A comprehensive breakdown of the sources from which studies were retrieved is presented in Supplementary Table S2. After removing duplicates and screening titles and abstracts for relevance, a total of 15 animal studies were included in the network meta-analysis [37,38,39,40,41,42,43,44,45,46,47,48,49,50,51]. Supplementary Table S3 lists the studies excluded during the final selection stage, along with specific reasons for their exclusion.

**Figure 2:**
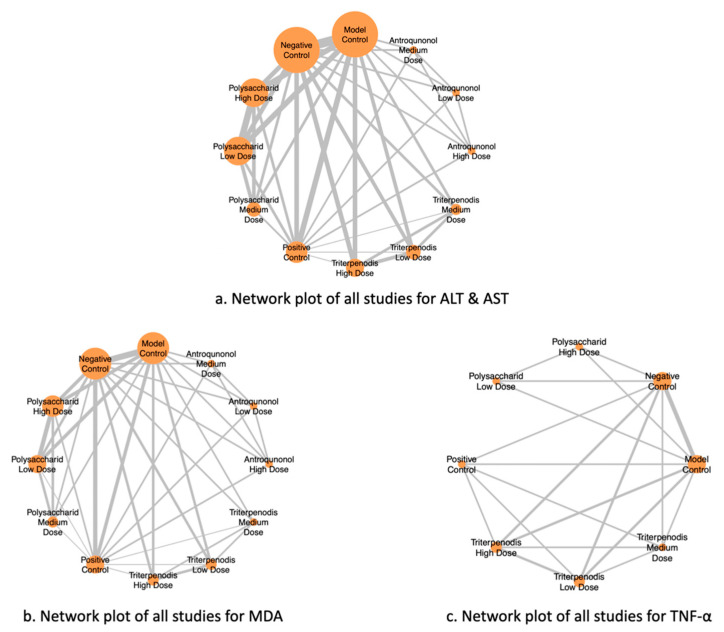
Network plots of all studies for liver function markers.

Figure 2 illustrates the NMA of different liver function markers, highlighting the comparisons between interventions. Nodes represent the intervention groups, with larger nodes indicating a greater number of studies evaluating each intervention. Edges (lines) represent direct comparisons between interventions, with thicker lines suggesting a greater number of included studies supporting that comparison. (a) ALT and AST: Network of studies assessing alanine aminotransferase (ALT) and aspartate aminotransferase (AST), key markers of liver function. (b) MDA: Network of studies evaluating malondialdehyde (MDA) levels, a biomarker of oxidative stress. (c) TNF-α: Network of studies analyzing tumor necrosis factor-alpha (TNF-α), an inflammatory cytokine linked to liver damage (see Figure 2a–c). This network structure enables an indirect comparison of interventions through shared comparators, enhancing the robustness of the meta-analysis.

Table 1 summarizes the characteristics of the included animal studies investigating the effects of *Antrodia cinnamomea* interventions on liver function and related biomarkers. It clearly presents the study authors and publication year and the specific animal models used (mouse or rat models) and provides detailed information about the intervention groups, including dosage regimens and group compositions. The control group setups are categorized as either negative/model controls or positive controls, allowing for straightforward comparisons. Key outcomes analyzed across studies are specifically focused on markers relevant to liver function (ALT, AST), oxidative stress (MDA), and inflammation (TNF-α). This selective and precise presentation ensures clarity and facilitates direct comparison, supporting subsequent network meta-analysis (see Table 1).

### 3.2. Methodological Quality of the Included Studies

The methodological quality of the 15 studies was assessed using SYRCLE’s risk of bias tool. For sequence generation, six studies (40.0%) were rated as low risk, and nine studies (60.0%) were rated as unclear risk. Regarding baseline characteristics, most studies (14 studies; 93.3%) were categorized as low risk, while one study (6.7%) was unclear risk. Allocation concealment and random housing were rated as unclear risk in all 15 studies (100%). Performance bias related to blinding was mostly unclear (13 studies; 86.7%), with two studies (13.3%) assessed as high risk. Random outcome assessment and detection bias (blinding) were also unclear risk across all studies (100%). All studies (100%) had low risk regarding incomplete outcome data and selective outcome reporting. For other biases, only three studies (20.0%) had low risk, whereas the remaining 12 studies (80.0%) were rated unclear risk. Overall, the assessment revealed a predominant presence of unclear risk of bias across the studies, as depicted in Figure S1, with detailed assessments available in Table S4.

### 3.3. Primary Outcome: High-Dose Triterpenoids Most Effective in Reducing ALT and Medium-Dose Triterpenoids Most Effective for AST

**Figure 3:**
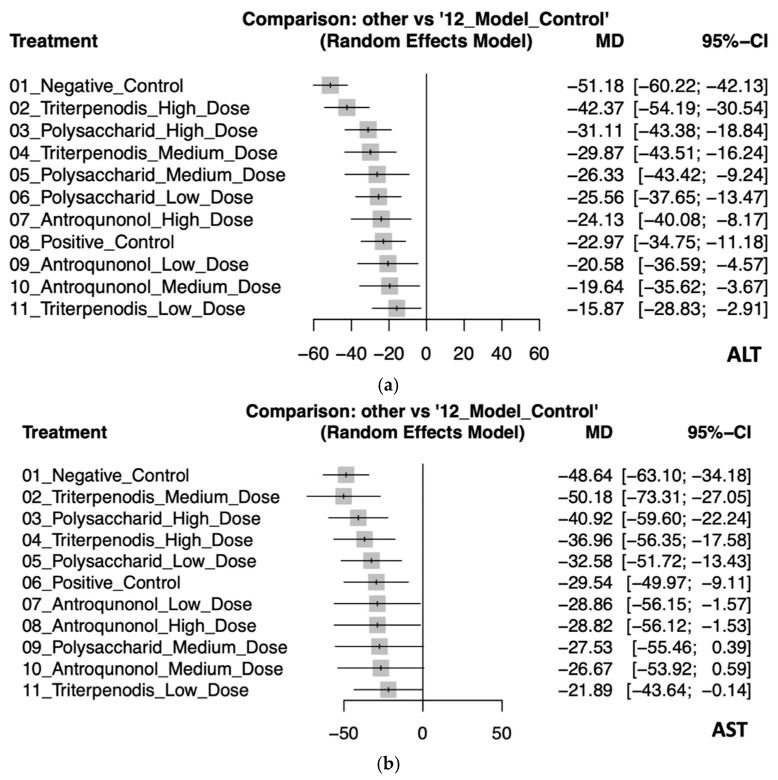
(**a**). Forest plot of the effects of *Antrodia cinnamomea* interventions on ALT levels in animal models. (**b**) Forest plot of the effects of *Antrodia cinnamomea* interventions on AST levels in animal models.

This NMA assessed the effectiveness of various treatments on liver enzyme ALT and AST levels compared to the model control group. For ALT levels, the negative control had the largest increase (MD: −51.18, 95% CI: −60.22 to −42.13), indicating the greatest deviation from the model control. High-dose triterpenoids demonstrated the most effective reduction (MD: −42.37, 95% CI: −54.19 to −30.54), followed by high-dose polysaccharides (MD: −31.11, 95% CI: −43.38 to −18.84). Medium-dose triterpenoids also showed a significant reduction (MD: −29.87, 95% CI: −43.51 to −16.24). The smallest reduction was observed with low-dose triterpenoids (MD: −15.87, 95% CI: −28.83 to −2.91), though still significant, reflecting some variability (see Figure 3a and Table 2a).

In the analysis for AST, medium-dose triterpenoids showed the most substantial effectiveness in enzyme reduction (MD: −50.18, 95% CI: −73.31 to −27.05), albeit with wide confidence intervals indicating uncertainty. High-dose polysaccharides (MD: −40.92, 95% CI: −59.60 to −22.24) and high-dose triterpenoids (MD: −36.96, 95% CI: −56.35 to −17.58) were also notably effective. The positive control and other interventions demonstrated varying degrees of enzyme reduction, with some interventions like medium-dose polysaccharides (MD: −27.53, 95% CI: −55.46 to 0.39) and medium-dose antroquinonol (MD: −26.67, 95% CI: −53.92 to 0.59) showing wide confidence intervals crossing zero, suggesting inconsistent effectiveness (see Figure 3b and Table 2b).

These findings highlight that higher-dose interventions, particularly triterpenoids, consistently provided significant and robust reductions in liver enzyme levels compared to controls. Detailed pairwise comparisons are further illustrated in Figure S2a,b.

### 3.4. Secondary Outcome: Antrodia cinnamomea Interventions Reduce Oxidative Stress and Inflammatory Cytokines

**Figure 4:**
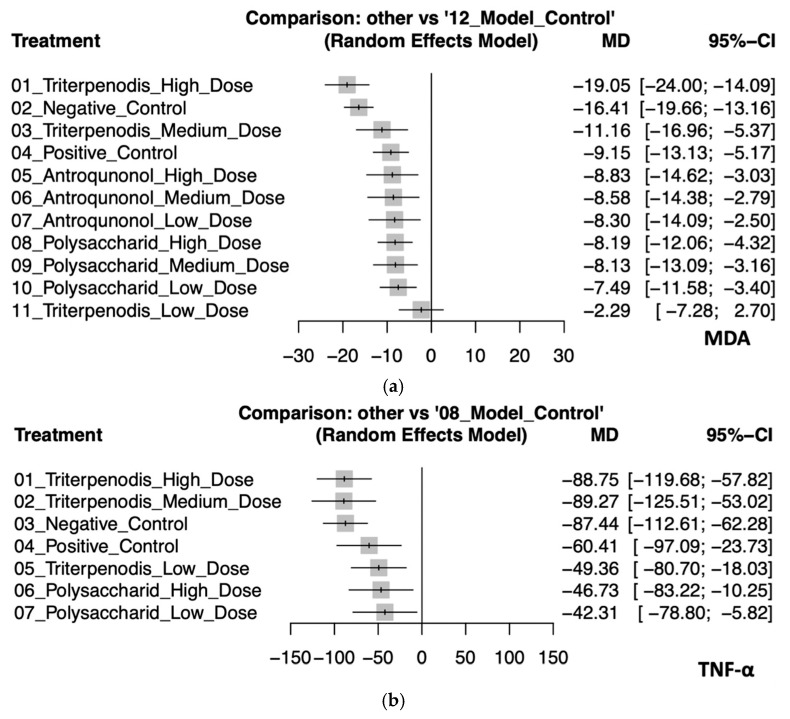
(**a**). Forest plot of the effects of *Antrodia cinnamomea* interventions on MDA levels in animal models. (**b**). Forest plot of the effects of *Antrodia cinnamomea* interventions on TNF-α levels in animal models.

This analysis evaluated the effects of various *Antrodia cinnamomea* interventions on oxidative stress (MDA) and inflammatory cytokines (TNF-α) in animal models. Regarding MDA, high-dose triterpenoid interventions demonstrated the most substantial reduction (MD: −19.05, 95% CI: −24.00 to −14.09). Negative control (MD: −16.41, 95% CI: −19.66 to −13.16), medium-dose triterpenoids (MD: −11.16, 95% CI: −16.96 to −5.37), and positive control (MD: −9.15, 95% CI: −13.13 to −5.17) also showed significant effects. Although interventions with high-dose (MD: −8.83; 95% CI: −14.62 to −3.03), medium-dose (MD: −8.58, 95% CI: −14.38 to −2.79), and low-dose (MD: −8.30, 95% CI: −14.09 to −2.50) antroquinonol and high (MD: −8.19, 95% CI: −12.06 to −4.32), medium (MD: −8.13, 95% CI: −13.09 to −3.16), and low doses (MD: −7.49, 95% CI: −11.58 to −3.40) of polysaccharide demonstrated moderate antioxidative properties, their confidence intervals substantially overlapped, which suggests minimal significant differences among these interventions. By contrast, low-dose triterpenoids (MD: −2.29, 95% CI: −7.28 to 2.70) had an inconclusive effect due to the CI crossing zero. For TNF-α, both high-dose (MD: −88.75, 95% CI: −119.68 to −57.82) and medium-dose (MD: −89.27, 95% CI: −125.51 to −53.02) triterpenoids exhibited the greatest anti-inflammatory effects, although their confidence intervals highly overlapped, which suggests similar efficacy. Negative control (MD: −87.44, 95% CI: −112.61 to −62.28) also closely followed. Interventions with positive control (MD: −60.41, 95% CI: −97.09 to −23.73), low-dose triterpenoids (MD: −49.36, 95% CI: −80.70 to −18.03), and high-dose (MD: −46.73, 95% CI: −83.22 to −10.25) and low-dose (MD: −42.31, 95% CI: −78.80 to −5.82) polysaccharides provided smaller yet significant reductions in TNF-α levels. The confidence intervals among these interventions overlapped, which indicates limited differentiation in the magnitude of effects. These findings collectively suggest that triterpenoid interventions, particularly at high and medium doses, possess superior efficacy in reducing both oxidative stress and inflammatory responses. Polysaccharide and antroquinonol extracts also demonstrated beneficial effects, with generally overlapping confidence intervals suggesting comparable yet slightly less potent effects. These trends reinforce the potential of *A. cinnamomea* bioactives in modulating liver-related oxidative and inflammatory pathways in preclinical models (see Figure 4a,b). Detailed pairwise comparisons among individual study arms are illustrated in Figure S3a,b.

### 3.5. Inconsistency Test

To evaluate the consistency of the NMA results, a node-splitting approach was conducted to compare the direct and indirect evidence for each intervention comparison. The analysis revealed significant inconsistency in some comparisons for ALT, particularly between the high-dose antroquinonol group and the model control group (difference: 145.04, 95% CI: 107.04 to 183.04, *p* < 0.01). However, most other comparisons demonstrated good consistency without significant discrepancies between direct and indirect estimates. For AST, significant inconsistency was observed in the comparison between high-dose antroquinonol and the model control (difference: 153.31, 95% CI: 88.46 to 218.16, *p* < 0.01), while most other comparisons showed acceptable consistency, suggesting the reliability of the network structure. Regarding MDA, significant inconsistency was identified specifically in the comparison between the high-dose antroquinonol group and the model control group (difference: 35.56, 95% CI: 21.95 to 49.17, *p* < 0.01). Nevertheless, other comparisons generally remained consistent, which indicates that most of the evidence was robust. For TNF-α, significant inconsistencies were noted in comparisons involving the high-dose polysaccharide group (difference: 231.89, 95% CI: 129.50 to 334.27, *p* < 0.01), the low-dose polysaccharide group (difference: 231.96, 95% CI: 129.54 to 334.38, *p* < 0.01), and the positive control group (difference: −326.26, 95% CI: −419.65 to −232.87, *p* < 0.01), all relative to the model control group. In summary, while most comparisons across outcomes demonstrated acceptable agreement between direct and indirect estimates, specific contrasts—particularly those involving high-dose antroquinonol and polysaccharide interventions—exhibited significant inconsistency. Accordingly, the findings from these comparisons should be interpreted with caution. Detailed results are provided in Table S5a–d.

### 3.6. Sensitivity Analyses

Sensitivity analyses using a leave-one-out approach were conducted for liver function biomarkers (ALT and AST) and for markers of oxidative stress and inflammation (MDA and TNF-α). The results for ALT and AST demonstrated overall consistency in the intervention effects. However, certain low-dose interventions (e.g., low-dose triterpenoids, low-dose antroquinonol) exhibited greater sensitivity to the exclusion of individual studies, which suggests potential instability in these estimates. For MDA, the effect of high-dose triterpenoids remained generally robust, although the exclusion of individual studies such as Cao et al. (2023) slightly affected the relative ranking of interventions [37]. For TNF-α, medium-dose triterpenoids occasionally ranked higher than high-dose triterpenoids; however, the effect sizes were comparable, with overlapping confidence intervals suggesting that both medium- and high-dose triterpenoids exert similarly favorable anti-inflammatory effects. See Figure S4a–d.

### 3.7. Publication Bias

Egger’s test was performed to assess the presence of potential publication bias across the four outcomes. The funnel plot for ALT showed no significant asymmetry (*p* = 0.28), which suggests the absence of publication bias. However, for AST, a statistically significant asymmetry was observed (*p* < 0.01), indicating possible publication bias. The results for MDA (*p* = 0.02) and TNF-α (*p* = 0.03) also suggested some degree of potential publication bias. These findings should be interpreted with caution and are illustrated in Figure S5a–d.

## 4. Discussion

### 4.1. Main Findings and Implications

To the best of our knowledge, this is the first NMA to evaluate the effects of *A. cinnamomea* on liver function biomarkers in animal studies. This study systematically compared the impact of various bioactive extracts of *A. cinnamomea*—including triterpenoids, polysaccharides, and antroquinonol—on commonly used biomarkers of hepatic injury (ALT, AST) and inflammation (MDA, TNF-α). The analysis revealed that triterpenoids consistently exhibited the most pronounced effects across all outcomes, significantly reducing ALT, AST, MDA, and TNF-α levels. Polysaccharides and antroquinonol also demonstrated beneficial but comparatively moderate effects, particularly in attenuating oxidative stress and inflammatory cytokines. These findings underscore the hepatoprotective potential of *A. cinnamomea* via multiple mechanisms and provide a scientific basis for its future development as a natural therapeutic agent.

### 4.2. Significance in the Context of Existing Research

Despite growing interest in the hepatoprotective activities of *A. cinnamomea*, prior research has largely focused on isolated bioactive components or singular mechanistic pathways without systematic quantitative comparisons [6,7]. For example, recent reviews such as Lin et al. (2025) highlighted the immunomodulatory and anticancer effects of polysaccharide fractions (Ac-GPS and Ac-SPS), yet they did not address the comparative efficacies among the different extract types or comprehensively assess dose-dependent impacts on standard hepatic biomarkers [11]. By contrast, our study employed an NMA approach that quantitatively integrates evidence across multiple extracts and doses, explicitly comparing their relative efficacies in modulating liver biomarkers (ALT, AST, MDA, and TNF-α). Notably, this analysis further clarifies the distinct biological roles of specific components previously described only qualitatively. While earlier studies identified triterpenoids as potent antioxidants [20,37,42], our findings provide direct quantitative evidence supporting their substantial role in reducing hepatic lipid peroxidation, reflected by significant declines in MDA. Moreover, although polysaccharides have been extensively studied for immunomodulatory functions [39,44,51], the current analysis quantitatively highlights their superior capacity in controlling inflammation via TNF-α suppression, reinforcing earlier mechanistic assertions about their cytokine regulatory roles. Collectively, this systematic and quantitative integration not only bridges gaps left by prior qualitative reviews but also advances a mechanistic understanding by linking quantitative biomarker improvements directly to specific bioactive components and their doses.

### 4.3. Possible Mechanistic Explanations

The significant reductions in ALT and AST levels observed in animals treated with triterpenoid-rich extracts suggest notable improvements in hepatocellular integrity and mitochondrial stability [20,37,42]. Mechanistically, triterpenoids exhibit bidirectional modulation of oxidative and inflammatory responses. They upregulate hepatic antioxidant enzymes, including superoxide dismutase (SOD), catalase (CAT), and glutathione peroxidase (GPx) [42,50], thereby reducing reactive oxygen species (ROS) and preventing hepatocellular necrosis and enzymatic leakage [37,50]. At the same time, they suppress pro-inflammatory pathways, potentially including NF-κB signaling. These antioxidant and anti-inflammatory activities are consistent with the marked reductions in MDA observed in our study, a well-established biomarker of lipid peroxidation and oxidative injury [25].

In addition, the substantial decreases in TNF-α levels associated with polysaccharide treatment observed in our analysis can be attributed to the anti-inflammatory properties of these extracts [44,51]. Polysaccharides and certain phenolic compounds derived from *A. cinnamomea* have been reported to suppress the production of pro-inflammatory cytokines, such as TNF-α and interleukin-6 (IL-6), by downregulating key inflammatory enzymes, including cyclooxygenase-2 (COX-2) and inducible nitric oxide synthase (iNOS) [47,51]. These actions may contribute to mild immunomodulation, potentially enhancing macrophage and natural killer (NK) cell activity, thereby enhancing immune surveillance and promoting tissue repair following hepatic injury [39,41].

*A. cinnamomea* may also exert metabolic benefits relevant to the progression of liver disease, particularly through the inhibition of fatty acid synthase (FAS), which reduces hepatic triglyceride accumulation and lipid droplet formation [38,43]. Although FAS inhibition was not directly measured in this study, prior evidence from animal models suggests that triterpenoids derived from *A. cinnamomea* suppress FAS activity [37,42]. Such metabolic regulation may partially account for the observed ALT, AST, and MDA reductions associated with high-dose triterpenoid extracts, possibly through a pathway involving reduced lipid peroxidation and lipid-induced cellular stress. Future investigations are warranted to clarify the relationship between FAS inhibition and hepatic enzyme normalization.

Moreover, antroquinonol may exert antifibrotic and cytoprotective effects by inhibiting hepatic stellate cell activation [45,50], as reported in previous studies showing its ability to reduce collagen deposition (e.g., type I collagen) [45]. This mechanism may complement the chronic liver injury-ameliorating effects of triterpenoid-rich extracts observed in our analysis, particularly the reduction in MDA levels. While antroquinonol may contribute to delaying fibrotic progression, triterpenoids may attenuate oxidative damage, suggesting a potentially synergistic dual action [37,42]. Although fibrotic markers were not directly assessed in our NMA, future studies should consider incorporating fibrosis-related biomarkers (e.g., collagen I, α-SMA) [5] to further elucidate the combined protective effects of *A. cinnamomea* extracts against both oxidative stress and fibrotic remodeling.

Notably, while high-dose triterpenoid extracts demonstrated substantial antioxidative efficacy in our analysis, potential concerns regarding dose-dependent cytotoxicity and safety in practical applications must be thoroughly addressed. Recent cell-based investigations have specifically evaluated the safety profile of high-dose *A. cinnamomea* triterpenoids, providing reassuring evidence of minimal cytotoxicity. Cheng et al. employed the MTT assay, a well-established method for assessing cell viability, across multiple cell lines, including human normal liver cells (LO2), human embryonic kidney cells (HEK293T), and hepatocellular carcinoma cells (HepG2). Their findings demonstrated negligible cytotoxicity even at concentrations substantially higher than the effective antioxidative doses, indicating an excellent safety margin [52]. The sustained high cell survival rates confirmed in these assays suggest that the potent hepatoprotective and antioxidant benefits observed at elevated dosages can indeed be achieved without substantial cytotoxic risks.

In summary, our findings suggest that triterpenoids reduce oxidative stress (MDA) and hepatic injury biomarkers (ALT, AST), while polysaccharides suppress inflammation (TNF-α) and enhance immune-mediated repair. Additional evidence suggests potential antifibrotic and metabolic regulatory effects. The multifaceted hepatoprotective effects of *A. cinnamomea* likely involve not only these antioxidative and anti-inflammatory mechanisms but also potential metabolic and fibrotic regulatory pathways, warranting further investigation.

### 4.4. Limitations

Despite the strengths of this analysis, several limitations should be acknowledged. First, like many NMAs involving preclinical studies, our analysis inevitably encountered heterogeneity due to differences in animal species, extraction methods, and dosing protocols across the included studies. This variability may have influenced intervention effects and complicated direct comparisons across studies. To address this, we deliberately restricted the inclusion criteria to rodent models and conducted stratified analyses by extract type and dose level. Although variations in dose classifications based on original study frameworks could lead to some potential bias, these stratifications significantly enhanced the interpretability and comparability of our findings. Second, as with much of the existing preclinical research, the methodological rigor varied among studies, with several lacking explicit reporting of randomization and blinding procedures. To transparently address this, we thoroughly assessed and reported the risk of bias using SYRCLE’s risk of bias tool. While potential biases due to unclear reporting cannot be fully excluded, our rigorous quality assessments and sensitivity analyses indicated that these issues did not substantially affect our main conclusions. Third, funnel plot analyses and Egger’s tests identified potential publication bias for certain biomarkers (AST, MDA, and TNF-α). While this suggests caution in interpreting the magnitude of some observed effects, our comprehensive sensitivity analyses confirmed the stability and overall robustness of these findings. Fourth, the diversity in the extraction methods and bioactive component profiles across studies might have introduced variability in treatment effects. Yet, this diversity reflects the real-world variability in practical usage and enhances the external validity of our findings. Future studies could further strengthen their evidence by standardizing extraction methods and explicitly characterizing bioactive components. Lastly, while results obtained from animal models do not translate directly into clinical guidelines, they provide the critical foundational evidence necessary for informing future clinical studies. Our findings offer clear and valuable directions for subsequent clinical translation and targeted therapeutic investigations.

## 5. Conclusions

This NMA demonstrates that *A. cinnamomea* extracts, particularly triterpenoids and polysaccharides, exhibit significant hepatoprotective effects. Triterpenoids were the most effective, significantly reducing hepatic injury biomarkers (ALT, AST), oxidative stress (MDA), and inflammatory cytokines (TNF-α). Polysaccharides also demonstrated significant anti-inflammatory and hepatoprotective effects. These findings are supported by consistent and statistically significant data, providing strong evidence for the potential of *A. cinnamomea* extracts as targeted hepatoprotective agents. Further clinical studies are warranted to confirm these benefits in human populations.

## Figures and Tables

**Table 1:** Summary of the effectiveness of *Antrodia cinnamomea* in improving liver function in animal models.

Study	Animal Model (n)	Intervention Groups	Control Groups	Outcomes
Cao et al., 2023 [37]	Mouse (C57BL/6) (*n* = 10/group)	ALI, Antcin A-L, Antcin A-H	Negative/Model	ALT, AST, MDA, TNF-α
Chyau et al., 2020 [38]	Mouse (*n* = 10/group)	HFD, Ant 40 mg/kg, HFD + Orlistat, HFD + Ant 20 mg/kg, HFD + Ant 40 mg/kg	Negative/Model/Positive	ALT, AST, MDA
Han et al., 2006 [39]	Mouse (*n* = 10/group)	*P. acnes* + LPS, ACN2a 0.2, 0.4, 0.8 g/kg, FK506 1 mg/kg	Negative/Model/Positive	ALT, AST
Ker et al., 2014 [40]	Rat (*n* = 6/group)	LPS, Antrodan control, Antrodan L + LPS, Antrodan H + LPS	Negative/Model/Positive	ALT, AST
Kim et al., 2024 [49]	Rat (*n* = 10/group)	Alcohol Control, A. camphorata 50, 100, 200 mg/kg, Silymarin 200 mg/kg)	Negative/Model/Positive	ALT, AST, MDA
Kumer et al., 2011 [50]	Mouse (*n* = 6/group)	Ethanol, EMAC 250 mg/kg, EMAC 500 mg/kg, EMAC 1000 mg/kg, Silymarin	Negative/Model/Positive	ALT, AST, MDA, TNF-α
Liu et al., 2017 [41]	Mouse (*n* = 24/group)	AC 0.1 g/kg, AC 0.3 g/kg, AC 0.9 g/kg	Negative/Model	ALT, AST, MDA
Liu et al., 2020 [42]	Mouse (*n* = 8/group)	Alcohol, Silibinin, ACT 5 mg/kg, ACT 15 mg/kg, ACT 45 mg/kg	Negative/Model/Positive	ALT, AST, TNF-α
Peng et al., 2017 [43]	Mouse (*n* = 10/group)	HFD, HFD + 0.5% ACE, HFD + 1% ACE, HFD + 2% ACE	Negative/Model	ALT, AST
Raun et al., 2022 [44]	Mouse (*n* = 10/group)	D-GalN/LPS, ACP 5 mg/kg, ACP 15 mg/kg	Negative/Model	ALT, AST, TNF-α
Shih et al., 2017 [45]	Rat (*n* = 10/group)	CCl_4_, Silymarin, AC 350 mg/kg, AC 1400 mg/kg, AC 3150 mg/kg	Negative/Model/Positive	ALT, AST, MDA
Wang et al., 2022 [46]	Mouse (*n* = 8/group)	Model, Low-dose, Medium-dose, High-dose	Negative/Model	ALT, AST, MDA
Wen et al., 2011 [47]	Mouse (*n* = 6/group)	Carrageenan, MEMAC 100, 200, 400 mg/kg, Indomethacin	Negative/Model/Positive	MDA, TNF-α
Xu et al., 2021 [48]	Mouse (*n* = 10/group)	Liver Injury Model, Bioactive Compound 100 mg/kg, 250 mg/kg, 500 mg/kg, Silymarin 50 mg/kg	Negative/Model	ALT, AST
Yang et al., 2022 [51]	Mouse (*n* = 10/group)	D-GalN/LPS, ACP 5 mg/kg, ACP 15 mg/kg	Negative/Model	ALT, AST, MDA, TNF-α

Abbreviations: ALT: Alanine aminotransferase, AST: Aspartate aminotransferase, MDA: Malondialdehyde, TNF-α: Tumor necrosis factor-alpha, LPS: Lipopolysaccharide, HFD: High-fat diet, CCl_4_: Carbon tetrachloride, D-GalN: D-Galactosamine, AC: *Antrodia cinnamomea*, ACN2a: *Antrodia cinnamomea* extract N2a, ACP: *Antrodia cinnamomea* polysaccharide, ACT: *Antrodia cinnamomea* triterpenoid, ACE: *Antrodia cinnamomea* ethanol extract, EMAC: Ethanolic extract from mycelia of *Antrodia cinnamomea*, MEMAC: Modified ethanolic extract from mycelia of *Antrodia cinnamomea*, ALI: Acute liver injury, Ant: Antroquinonol.

**Table 2:** a. The results for ALT from pairwise meta-analyses (above the diagonal) and network meta-analysis (below the diagonal). Effect sizes are presented as mean differences (MDs) with corresponding 95% confidence intervals (CIs), allowing for comparison across all interventions. b. Results for AST from pairwise meta-analyses (above the diagonal) and network meta-analysis (below the diagonal). Effect sizes are presented as mean differences (MDs) with corresponding 95% confidence intervals (CIs), allowing for comparison across all interventions.

a.												
	Negative Control	Triterpenoids High Dose	Polysaccharides High Dose	Triterpenoids Medium Dose	Polysaccharides Medium Dose	Polysaccharides Low Dose	Antroquinonol High Dose	Positive Control	Antroquinonol Low Dose	Antroquinonol Medium Dose	Triterpenoids Low Dose	Model Control
Negative Control	Negative Control	−11.34 [−23.60; 0.92]	−27.77 [−43.75; −11.79]	−10.00 [−25.27; 5.28]	−7.07 [−33.24; 19.10]	−40.97 [−56.69; −25.24]	10.57 [−7.91; 29.04]	2.78 [−12.17; 17.73]	7.03 [−11.49; 25.55]	6.17 [−12.32; 24.66]	−34.96 [−49.01; −20.91]	−54.85 [−64.93; −44.77]
Triterpenoids High Dose	−8.81 [−20.25; 2.64]	Triterpenoids High Dose	−	−5.62 [−20.96; 9.73]	−	−	−	0.43 [−25.21; 26.06]	−	−	−21.03 [−35.14; −6.92]	−49.23 [−62.34; −36.13]
Polysaccharides High Dose	−20.06 [−31.79; −8.33]	−11.26 [−26.59; 4.08]	Polysaccharides High Dose	−	0.04 [−18.44; 18.51]	−9.21 [−21.55; 3.14]	−	−13.40 [−39.90; 13.11]	−	−	−	−52.16 [−68.46; −35.86]
Triterpenoids Medium Dose	−21.30 [−34.77; −7.83]	−12.49 [−26.83; 1.84]	−1.24 [−18.06; 15.58]	Triterpenoids Medium Dose	−	−	−	0.40 [−25.23; 26.03]	−	−	−10.08 [−25.31; 5.15]	−16.67 [−32.17; −1.16]
Polysaccharides Medium Dose	−24.84 [−41.68; −8.01]	−16.04 [−35.53; 3.45]	−4.78 [−21.29; 11.73]	−3.54 [−24.23; 17.14]	Polysaccharides Medium Dose	−3.10 [−21.48; 15.29]	−	299.20 [−35.28; 633.67]	−	−	−	−7.25 [−33.51; 19.02]
Polysaccharides Low Dose	−25.62 [−37.20; −14.04]	−16.81 [−32.01; −1.61]	−5.56 [−17.25; 6.14]	−4.32 [−21.02; 12.38]	−0.78 [−17.22; 15.67]	Polysaccharides Low Dose	−	−1.40 [−27.66; 24.86]	−	−	−	−36.07 [−51.98; −20.16]
Antroquinonol High Dose	−27.05 [−43.10; −11.00]	−18.24 [−36.77; 0.29]	−6.98 [−25.68; 11.71]	−5.75 [−25.48; 13.98]	−2.20 [−24.48; 20.08]	−1.43 [−20.02; 17.16]	Antroquinonol High Dose	0.66 [−17.57; 18.88]	−3.55 [−21.81; 14.71]	−4.49 [−22.71; 13.74]	−	9.01 [−9.15; 27.18]
Positive Control	−28.21 [−40.16; −16.26]	−19.40 [−34.00; −4.81]	−8.15 [−22.79; 6.49]	−6.91 [−22.87; 9.05]	−3.37 [−22.54; 15.81]	−2.59 [−17.10; 11.92]	−1.16 [−17.74; 15.41]	Positive Control	−4.21 [−22.48; 14.07]	−5.14 [−23.38; 13.10]	−0.81 [−26.47; 24.85]	−2.33 [−15.34; 10.68]
Antroquinonol Low Dose	−30.59 [−46.70; −14.48]	−21.78 [−40.36; −3.21]	−10.53 [−29.27; 8.21]	−9.29 [−29.07; 10.48]	−5.75 [−28.07; 16.57]	−4.97 [−23.61; 13.66]	−3.55 [−21.80; 14.71]	−2.38 [−19.01; 14.25]	Antroquinonol Low Dose	−0.92 [−19.19; 17.36]	−	12.52 [−5.70; 30.74]
Antroquinonol Medium Dose	−31.53 [−47.60; −15.46]	−22.72 [−41.26; −4.18]	−11.47 [−30.17; 7.24]	−10.23 [−29.97; 9.51]	−6.69 [−28.98; 15.60]	−5.91 [−24.51; 12.69]	−4.48 [−22.71; 13.74]	−3.32 [−19.91; 13.27]	−0.94 [−19.21; 17.34]	Antroquinonol Medium Dose	−	13.50 [−4.68; 31.68]
Triterpenoids Low Dose	−35.31 [−48.03; −22.58]	−26.50 [−40.08; −12.91]	−15.24 [−31.50; 1.02]	−14.00 [−28.82; 0.81]	−10.46 [−30.69; 9.77]	−9.69 [−25.82; 6.45]	−8.26 [−27.52; 11.01]	−7.10 [−22.53; 8.34]	−4.71 [−24.03; 14.60]	−3.77 [−23.06; 15.51]	Triterpenoids Low Dose	−13.57 [−28.15; 1.01]
Model Control	−51.18 [−60.22; −42.13]	−42.37 [−54.19; −30.54]	−31.11 [−43.38; −18.84]	−29.87 [−43.51; −16.24]	−26.33 [−43.42; −9.24]	−25.56 [−37.65; −13.47]	−24.13 [−40.08; −8.17]	−22.97 [−34.75; −11.18]	−20.58 [−36.59; −4.57]	−19.64 [−35.62; −3.67]	−15.87 [−28.83; −2.91]	Model Control
b.												
	Negative Control	Triterpenoids Medium Dose	Polysaccharides High Dose	Triterpenoids High Dose	Polysaccharides Low Dose	Positive Control	Antroquinonol Low Dose	Antroquinonol High Dose	Polysaccharides Medium Dose	Antroquinonol Medium Dose	Triterpenoids Low Dose	Model Control
Negative Control	Negative Control	1.91 [−24.02; 27.84]	−18.61 [−43.86; 6.64]	−14.82 [−35.18; 5.54]	−23.33 [−48.98; 2.32]	2.76 [−19.28; 24.80]	21.40 [−10.04; 52.85]	21.38 [−10.06; 52.82]	−26.92 [−70.12; 16.28]	19.29 [−12.13; 50.70]	−31.84 [−55.33; −8.35]	−47.17 [−63.32; −31.02]
Triterpenoids Medium Dose	1.54 [−21.21; 24.28]	Triterpenoids Medium Dose	−	−21.32 [−47.42; 4.77]	−	−0.10 [−44.17; 43.97]	−	−	−	−	−23.82 [−50.95; 3.32]	−40.81 [−67.83; −13.80]
Polysaccharides High Dose	−7.72 [−26.56; 11.12]	−9.26 [−36.93; 18.41]	Polysaccharides High Dose	−	−11.65 [−31.60; 8.29]	−25.66 [−69.14; 17.82]	−	−	−3.36 [−34.17; 27.46]	−	−	−58.73 [−82.13; −35.34]
Triterpenoids High Dose	−11.68 [−30.62; 7.27]	−13.22 [−37.44; 11.01]	−3.96 [−28.63; 20.72]	Triterpenoids High Dose	−	2.86 [−41.35; 47.07]	−	−	−	−	−9.44 [−33.41; 14.54]	−43.37 [−64.53; −22.21]
Polysaccharides Low Dose	−16.06 [−35.27; 3.14]	−17.60 [−45.64; 10.43]	−8.35 [−27.25; 10.56]	−4.39 [−29.45; 20.67]	Polysaccharides Low Dose	415.97 [−53.09; 885.04]	−	−	3.76 [−27.12; 34.64]	−	−	−42.65 [−66.10; −19.20]
Positive Control	−19.10 [−38.91; 0.70]	−20.64 [−47.92; 6.64]	−11.38 [−35.46; 12.70]	−7.43 [−32.05; 17.20]	−3.04 [−28.27; 22.20]	Positive Control	−4.47 [−35.59; 26.66]	−4.51 [−35.63; 26.61]	−312.33 [−707.93; 83.27]	−6.69 [−37.78; 24.40]	3.56 [−40.51; 47.63]	−3.03 [−28.34; 22.29]
Antroquinonol Low Dose	−19.78 [−46.97; 7.41]	−21.32 [−55.02; 12.39]	−12.06 [−43.12; 19.01]	−8.10 [−39.48; 23.27]	−3.71 [−35.25; 27.83]	−0.68 [−29.04; 27.69]	Antroquinonol Low Dose	−0.04 [−31.26; 31.18]	−	−2.20 [−33.39; 28.99]	−	6.39 [−24.71; 37.50]
Antroquinonol High Dose	−19.82 [−47.01; 7.38]	−21.36 [−55.06; 12.35]	−12.10 [−43.16; 18.97]	−8.14 [−39.52; 23.24]	−3.75 [−35.29; 27.79]	−0.71 [−29.08; 27.65]	−0.04 [−31.26; 31.18]	Antroquinonol High Dose	−	−2.16 [−33.35; 29.03]	−	6.44 [−24.66; 37.54]
Polysaccharides Medium Dose	−21.11 [−49.06; 6.85]	−22.64 [−57.27; 11.98]	−13.39 [−40.67; 13.89]	−9.43 [−41.69; 22.83]	−5.04 [−32.42; 22.34]	−2.00 [−34.35; 30.34]	−1.33 [−38.83; 36.18]	−1.29 [−38.79; 36.22]	Polysaccharides Medium Dose	−	−	−15.37 [−58.90; 28.16]
Antroquinonol Medium Dose	−21.97 [−49.13; 5.18]	−23.51 [−57.19; 10.17]	−14.25 [−45.29; 16.78]	−10.30 [−41.64; 21.05]	−5.91 [−37.42; 25.60]	−2.87 [−31.20; 25.46]	−2.20 [−33.38; 28.99]	−2.16 [−33.35; 29.03]	−0.87 [−38.35; 36.61]	Antroquinonol Medium Dose	−	8.63 [−22.44; 39.70]
Triterpenoids Low Dose	−26.75 [−48.09; −5.41]	−28.29 [−53.91; −2.67]	−19.03 [−45.57; 7.51]	−15.07 [−38.10; 7.95]	−10.68 [−37.60; 16.23]	−7.65 [−33.91; 18.62]	−6.97 [−39.79; 25.84]	−6.93 [−39.75; 25.88]	−5.64 [−39.36; 28.08]	−4.78 [−37.56; 28.01]	Triterpenoids Low Dose	−19.54 [−43.85; 4.77]
Model Control	−48.64 [−63.10; −34.18]	−50.18 [−73.31; −27.05]	−40.92 [−59.60; −22.24]	−36.96 [−56.35; −17.58]	−32.58 [−51.72; −13.43]	−29.54 [−49.97; −9.11]	−28.86 [−56.15; −1.57]	−28.82 [−56.12; −1.53]	−27.53 [−55.46; 0.39]	−26.67 [−53.92; 0.59]	−21.89 [−43.64; −0.14]	Model Control

## Data Availability

The data are included in the article and the Supplementary Materials.

## Supplementary Materials

The following supporting information can be downloaded at: https://www.mdpi.com/article/10.3390/antiox14060660/s1, Table S1: PRISMA for NMA checklist, Table S2: Keywords and search results in different databases, Table S3: Studies excluded from the analysis, along with the reasons for their exclusion, Table S4: Risk of bias assessment of 15 studies using SYRCLE’s tool, Table S5: Inconsistency test outcomes; Figure S1: SYRCLE’s Risk of bias tool, Figure S2: Individual study results (a: ALT, b: AST), Figure S3: Individual study results (a: MDA, b: TNF-α), Figure S4: The forest plots of sensitivity analysis (leave-one-out), Figure S5: Publication bias assessed by Egger’s test.
