# Supplementary material for: Comparative Efficacy of Antrodia cinnamomea on Liver Function Biomarkers in Mice and Rats: A Network Meta-Analysis
Source: Antioxidants (Basel). 2025 May 30;14(6):660. doi: 10.3390/antiox14060660 (PMC12189407; doi:10.3390/antiox14060660)
Supplement: Supplementary file 1 [file antioxidants-14-00660-s001.zip › antioxidants-3633830-supplementary.pdf]

Table S1 - PRISMA for network meta-analysis checklist

Section and Topic	#	Checklist item	Location
**Title**			
Title	1	Identify the report as a systematic review incorporating a network meta-analysis (or related form of meta-analysis).	Title
**Abstract**			
Structured summary	2	Provide a structured summary including, as applicable: Background: main objectives / Methods: data sources; study eligibility criteria, participants, and interventions; study appraisal; and synthesis methods, such as network meta-analysis. / Results: number of studies and participants identified; summary estimates with corresponding confidence/credible intervals; treatment rankings may also be discussed. Authors may choose to summarize pairwise comparisons against a chosen treatment included in their analyses for brevity. / Discussion/conclusions: limitations; conclusions and implications of findings. / Other: primary source of funding; systematic review registration number with registry name.	Abstract
**Introduction**			
Rationale	3	Describe the rationale for the review in the context of what is already known, including mention of why a network meta-analysis has been conducted.	Introduction / 1st and 2nd paragraph
Objectives	4	Provide an explicit statement of questions being addressed, with reference to participants, interventions, comparisons, outcomes, and study design (PICOS).	Introduction / 3rd paragraph
**Methods**			
Protocol & registration	5	Indicate whether a review protocol exists and where it can be accessed; and, if available, provide registration information, including registration number.	Materials and Methods/ 1st paragrph
Eligibility criteria	6	Specify study characteristics (e.g., PICOS, length of follow-up) and report characteristics (e.g., years considered, language, publication status) used as criteria for eligibility, giving	Materials and Methods/ Inculusion and exculusion

		rationale. Clearly describe eligible treatments included in the treatment network, and note whether any have been clustered or merged into the same node (with justification).	
Information sources	7	Describe all information sources (e.g., databases with dates of coverage, contact with study authors) in the search and date last searched.	Table S2
Search	8	Present full electronic search strategy for at least one database, including any limits used, such that it could be repeated.	Table S2
Study selection	9	State the process for selecting studies (i.e., screening, eligibility, included in systematic review, and, if applicable, included in the meta-analysis).	Materials and Methods / Study Identification / Inculusion and exculusion criteria
Data collection	10	Describe method of data extraction from reports (e.g., piloted forms, independently, in duplicate) and any processes for obtaining and confirming data.	Materials and Methods / Data extraction
Data items	11	List and define all variables for which data were sought (e.g., PICOS, funding sources) and any assumptions and simplifications made.	Materials and Methods / Data extraction and conversion
Network geometry	S1	Describe methods used to explore the geometry of the treatment network under study and potential biases related to it. This should include how the evidence base has been graphically summarized for presentation, and what characteristics were compiled and used to describe the evidence base to readers.	Materials and Methods / Modeling for network meta-analysis
Risk of bias within	12	Describe methods used for assessing risk of bias of individual studies (including specification of whether this was done at the study or outcome level), and how this information is to be used in any data synthesis.	Materials and Methods / Quality appraisal
Summary measures	13	State the principal summary measures (e.g., risk ratio, difference in means). Also describe the use of additional summary measures assessed, such as treatment rankings, as well as modified approaches used to present summary findings from meta-analyses.	Materials and Methods / Outcome
Planned methods of analysis	14	Describe the methods of handling data and combining results of studies for each network meta-analysis. This should include, but not be limited to: Handling of multi-arm trials;	Materials and Methods / Statistical analyses

		Selection of variance structure; Selection of prior distributions in Bayesian analyses; and Assessment of model fit.	
Assessment of inconsistency	S2	Describe the statistical methods used to evaluate the agreement of direct and indirect evidence in the treatment network(s) studied. Describe efforts taken to address its presence when found.	Materials and Methods / Statistical analyses
Risk of bias across	15	Specify any assessment of risk of bias that may affect the cumulative evidence.	Materials and Methods / Publication bias
Additional analyses	16	Describe methods of additional analyses if done, indicating which were pre-specified. This may include, but not be limited to, the following: Sensitivity or subgroup analyses; Meta-regression analyses; Alternative formulations of the treatment network; and Use of alternative prior distributions for Bayesian analyses (if applicable).	Materials and Methods / Sensitivity analyses
**Results**			
Study selection	17	Give numbers of studies screened, assessed for eligibility, and included in the review, with reasons for exclusions at each stage, ideally with a flow diagram.	Results / Study identification Figure 1, Table S2, Table S3
Network structure	S3	Provide a network graph of the included studies to enable visualization of the geometry of the treatment network.	Fugre 2
Network geometry	S4	Provide a brief overview of characteristics of the treatment network. This may include commentary on the abundance of trials and randomized patients for the different interventions and pairwise comparisons in the network, gaps of evidence in the treatment network, and potential biases reflected by the network structure.	Results / Network model formation / Figure 2
Study characteristics	18	For each study, present characteristics for which data were extracted (e.g., study size, PICOS, follow-up period) and provide the citations.	Table 1
Risk of bias within	19	Present data on risk of bias of each study and, if available, any outcome level assessment.	Table S4, Figure S1, Methodological quality

Results of individual studies	20	For all outcomes considered (benefits or harms), present, for each study: (1) simple summary data for each intervention group, and (2) effect estimates and confidence intervals. Modified approaches may be needed to deal with information from larger networks.	Table 1
Synthesis of results	21	Present results of each meta-analysis done, including confidence/credible intervals. In larger networks, authors may focus on comparisons versus a particular comparator (e.g. placebo or standard care), with full findings presented in an appendix. League tables and forest plots may be considered to summarize pairwise comparisons. If additional summary measures were explored (such as treatment rankings), these should also be presented.	Outcomes / Figure 3, Figure 4, Figure S2, Figure S3, Table 2
Exploration for inconsistency	S5	Describe results from investigations of inconsistency. This may include such information as measures of model fit to compare consistency and inconsistency models, P values from statistical tests, or summary of inconsistency estimates from different parts of the treatment network.	Inconsistency test<br>Table S5
Risk of bias across	22	Present results of any assessment of risk of bias across studies for the evidence base being studied.	Publicaton bias, Figure S1
Additional analyses	23	Give results of additional analyses, if done (e.g., sensitivity or subgroup analyses, meta-regression analyses, alternative network geometries studied, alternative choice of prior distributions for Bayesian analyses, and so forth).	Sensitivity analysis / Figure S4a-d
**Discussion**			
Summary of evidence	24	Summarize the main findings, including the strength of evidence for each main outcome; consider their relevance to key groups.	Discussion<br>Findings and implications
Limitations	25	Discuss limitations at study and outcome level (e.g., risk of bias), and at review level (e.g., incomplete retrieval of identified research, reporting bias). Comment on the validity of the assumptions, such as transitivity and consistency. Comment on any concerns regarding network geometry (e.g., avoidance of certain comparisons).	Discussion<br>Limitations

Conclusions	26	Provide a general interpretation of the results in the context of other evidence, and implications for future research.	Conclusion
**Funding**			
Funding	27	Describe sources of funding for the systematic review and other support (e.g., supply of data); role of funders for the systematic review. This should also include information regarding whether funding has been received from manufacturers of treatments in the network and/or whether some of the authors are content experts with professional conflicts of interest that could affect use of treatments in the network.	Funding

Table S2 - Keywords and search results in different databases ***770***

Database	Keyword	Date	Results
PubMed	antrodia cinnamomea AND hepatoprotection	2024.11.29	37
Embase	antrodia cinnamomea AND hepatoprotection	2024.11.29	56
Cochrane CENTRAL	antrodia cinnamomea AND hepatoprotection	2024.11.29	2
Web of Science	antrodia cinnamomea AND hepatoprotection	2024.11.29	9
			104

Database	Keyword	Date	Results
PubMed	antrodia cinnamomea AND Non-Alcoholic Fatty Liver Disease	2024.11.29	6
Embase	antrodia cinnamomea AND Non-Alcoholic Fatty Liver Disease	2024.11.29	3
Cochrane CENTRAL	antrodia cinnamomea AND Non-Alcoholic Fatty Liver Disease	2024.11.29	1
Web of Science	antrodia cinnamomea AND Non-Alcoholic Fatty Liver Disease	2024.11.29	3
			13

Database	Keyword	Date	Results
PubMed	antrodia cinnamomea OR antrodia camphoratus OR Taiwanofungus camphoratus AND triterpenoids AND liver	2024.11.29	21
Embase	antrodia cinnamomea OR antrodia camphoratus OR Taiwanofungus camphoratus AND triterpenoids AND liver	2024.11.29	56
Cochrane CENTRAL	antrodia cinnamomea OR antrodia camphoratus OR Taiwanofungus camphoratus AND triterpenoids AND liver	2024.11.29	11
Web of Science	antrodia cinnamomea OR antrodia camphoratus OR Taiwanofungus camphoratus AND triterpenoids AND liver	2024.11.29	530

Database	Keyword	Date	Results
PubMed	antrodia cinnamomea AND polysaccharids AND liver	2024.11.29	17
Embase	antrodia cinnamomea AND polysaccharids AND liver	2024.11.29	0
Cochrane CENTRAL	antrodia cinnamomea AND polysaccharids AND liver	2024.11.29	0
Web of Science	antrodia cinnamomea AND polysaccharids AND liver		0
			17

Database	Keyword	Date	Results
PubMed	antrodia cinnamomea AND β-Glucan AND liver	2024.11.29	1
Embase	antrodia cinnamomea AND β-Glucan AND liver	2024.11.29	1
Cochrane CENTRAL	antrodia cinnamomea AND β-Glucan AND liver	2024.11.29	0
Web of Science	antrodia cinnamomea AND β-Glucan AND liver	2024.11.29	1
			3

Database	Keyword	Date	Results
PubMed	antrodia cinnamomea AND antrodin AND liver	2024.11.29	1
Embase	antrodia cinnamomea AND antrodin AND liver	2024.11.29	1
Cochrane CENTRAL	antrodia cinnamomea AND antrodin AND liver	2024.11.29	0
Web of Science	antrodia cinnamomea AND antrodin AND liver	2024.11.29	1
			3

Database	Keyword	Date	Results
PubMed	antrodia cinnamomea AND antroquinonol AND liver	2024.11.29	4
Embase	antrodia cinnamomea AND antroquinonol AND liver	2024.11.29	5
Cochrane CENTRAL	antrodia cinnamomea AND antroquinonol AND liver	2024.11.29	0
Web of Science	antrodia cinnamomea AND antroquinonol AND liver	2024.11.29	3
			12

Database	Keyword	Date	Results
PubMed	antrodia cinnamomea AND flavoids AND liver	2024.11.29	0
Embase	antrodia cinnamomea AND flavoids AND liver	2024.11.29	0
Cochrane CENTRAL	antrodia cinnamomea AND flavoids AND liver	2024.11.29	0
Web of Science	antrodia cinnamomea AND flavoids AND liver	2024.11.29	0
			0

Database	Keyword	Date	Results
PubMed	antrodia cinnamomea AND exsosome AND liver	2024.11.29	0
Embase	antrodia cinnamomea AND exsosome AND liver	2024.11.29	0
Cochrane CENTRAL	antrodia cinnamomea AND exsosome AND liver	2024.11.29	0
Web of Science	antrodia cinnamomea AND exsosome AND liver	2024.11.29	0
			0

Database	Keyword	Date	Results

PubMed	antrodia cinnamomea AND benzoids AND liver	2024.11.29	0
Embase	antrodia cinnamomea AND benzoids AND liver	2024.11.29	0
Cochrane CENTRAL	antrodia cinnamomea AND benzoids AND liver	2024.11.29	0
Web of Science	antrodia cinnamomea AND benzoids AND liver	2024.11.29	0
			0

Database	Keyword	Date	Results
PubMed	antrodia cinnamomea AND exopolysaccharids AND liver	2024.11.29	0
Embase	antrodia cinnamomea AND exopolysaccharids AND liver	2024.11.29	0
Cochrane CENTRAL	antrodia cinnamomea AND exopolysaccharids AND liver	2024.11.29	0
Web of Science	antrodia cinnamomea AND exopolysaccharids AND liver	2024.11.29	0
			0

Database	Keyword	Date	Results
PubMed	antrodia cinnamomea AND polyphenols AND liver	2024.11.29	0
Embase	antrodia cinnamomea AND polyphenols AND liver	2024.11.29	0
Cochrane CENTRAL	antrodia cinnamomea AND polyphenols AND liver	2024.11.29	0
Web of Science	antrodia cinnamomea AND polyphenols AND liver	2024.11.29	0
			0

Table S3: Studies excluded from the analysis along with the reasons for their exclusion

No.	First Author / Publication Year/ Title		Journal/Book	Exclusion reasons
1	Chen et al., 2018	Anti-Metastatic Effects of Antrodan with and without Cisplatin on Lewis Lung Carcinomas in a Mouse Xenograft Model	Int J Mol Sci	Unrelated
2	Cheng et al., 2022	Dehydroeburicoic Acid, a Dual Inhibitor against Oxidative Stress in Alcoholic Liver Disease	Pharmaceuticals (Basel)	Non-animal studies
3	Cheng et al., 2023	Cell study (in vitro, ALD model)		Non-animal studies
4	Chiou et al., 2021	Hepatoprotective Effect of Antrodia cinnamomea Mycelium in Patients with Nonalcoholic Steatohepatitis: A Randomized, Double-Blind, Placebo-Controlled Trial	J Am Coll Nutr	Non-mouse or non-rat animal model
5	Cao et al., 2022	Antrodia cinnamomea and its compound dehydroeburicoic acid attenuate nonalcoholic fatty liver disease by upregulating ALDH2 activity	J Ethnopharmacol	No relevant outcome data
6	Chou et al., 2019	4-Hydroxybenzoic acid serves as an endogenous ring precursor for antroquinonol biosynthesis in Antrodia cinnamomea		Unrelated
7	Huang et al., 2015	Antcin K, an Active Triterpenoid from the Fruiting Bodies of Basswood-Cultivated Antrodia cinnamomea, Inhibits Metastasis via Suppression of Integrin-Mediated Adhesion, Migration, and Invasion in Human Hepatoma Cells	J Agric Food Chem	Non-animal studies
8	Kong et al., 2020	Nanoparticles of Antroquinonol-Rich Extract from Solid-State-Cultured Antrodia cinnamomea Improve Reproductive Function in Diabetic Male Rats		Unrelated
9	Kuang et al., 2021	Terpenoids from the medicinal mushroom Antrodia camphorata: chemistry and medicinal potential	Nat Prod Rep	Review
10	Lai et al., 2015	Antcin K, an active triterpenoid from the fruiting bodies of basswood cultivated Antrodia cinnamomea, induces mitochondria and endoplasmic reticulum stress-mediated apoptosis in human hepatoma cells	J Tradit Complement Med	Non-animal studies

11	Lee et al., 2018	Evaluation of potential antioxidant and anti-inflammatory effects of Antrodia cinnamomea powder and the underlying molecular mechanisms via Nrf2- and NF-κB-dominated pathways in broiler chickens	Poult Sci	Unrelated
12	Li et al., 2023	Application of response surface methodology and quantitative NMR for the optimum extraction, characterization, and quantitation of Antrodia cinnamomea triterpenoids	Sci Rep	Unrelated
13	Lien et al., 2014	Comparison of the apoptotic effects of supercritical fluid extracts of Antrodia cinnamomea mycelia on hepatocellular carcinoma cells	Molecules	Non-animal studies
14	Lin et al., 2015	The Ethanolic Extract of Taiwanofungus camphoratus (Antrodia camphorata) Induces Cell Cycle Arrest and Enhances Cytotoxicity of Cisplatin and Doxorubicin on Human Hepatocellular Carcinoma Cells	Biomed Res Int	Non-animal studies
15	Lin et al., 2025	Anti-inflammatory and anticancer effects of polysaccharides from Antrodia cinnamomea : A review	J Chin Med Assoc	Review
16	Liu et al., 2023	A review on the protective effect of active components in Antrodia camphorata against alcoholic liver injury	J Ethnopharmacol	Review
17	Ma et al., 2024	Antrodia cinnamomea triterpenoids attenuate cardiac hypertrophy via the SNW1/RXR/ALDH2 axis	Redox Biol	Unrelated
18	Perera et al., 2018	Antrodia cinnamomea Galactomannan Elicits Immuno-stimulatory Activity Through Toll-like Receptor 4	Int J Biol Sci	Non-animal studies
19	Su et al., 2012	Eburicoic Acid, an Active Triterpenoid from the Fruiting Bodies of Basswood Cultivated Antrodia cinnamomea, Induces ER Stress-Mediated Autophagy in Human Hepatoma Cells	J Tradit Complement Med	Non-animal studies
20	Wang et al., 2013	Establishment of the metabolite profile for an Antrodia cinnamomea health food product and investigation of its chemoprevention activity	J Agric Food Chem	Unrelated
21	Wang et al., 2019	Antcins, triterpenoids from Antrodia cinnamomea, as new agonists for peroxisome proliferator-activated receptor α	J Food Drug Anal	Unrelated

22	Wu et al., 2016	Extracted Triterpenes from Antrodia cinnamomea Reduce the Inflammation to Promote the Wound Healing via the STZ Inducing Hyperglycemia-Diabetes Mice Model	Front Pharmacol	Unrelated
23	Wu et al., 2018	Antrodia cinnamomea boosts the anti-tumor activity of sorafenib in xenograft models of human hepatocellular carcinoma	Sci Rep	Non-animal studies
24	Yang et al., 2009	Anti-angiogenic effects and mechanisms of polysaccharides from Antrodia cinnamomea with different molecular weights	J Ethnopharmacol	Unrelated
25	Ye et al., 2022	Antrodia cinnamomea polysaccharide improves liver antioxidant, anti-inflammatory capacity, and cecal flora structure of slow-growing broiler breeds challenged with lipopolysaccharide	Front Vet Sci	Non-mouse or non-rat animal model

Table S4: Risk of bias assessment of 15 studies using SYRCLE's tool

	Selection bias - Sequence generation	Selection bias - Baseline characteristics	Selection bias - Allocation concealment	Performance bias - Random housing	Performance bias - Blinding	Detection bias - Random outcome assessment	Detection bias - Blinding	Attrition bias - Incomplete outcome data	Reporting bias - Selective outcome reporting	Other biases - Other sources of biases
Cao et al., 2023	L	L	S	S	S	S	S	L	L	S
Chyau et al., 2020	S	L	S	S	H	S	S	L	L	S
Han et al., 2006	S	L	S	S	S	S	S	L	L	S
Ker et al., 2014	L	L	S	S	S	S	S	L	L	L
Kim et al., 2024	S	L	S	S	S	S	S	L	L	S
Kumer et al., 2011	S	S	S	S	S	S	S	L	L	S
Liu et al., 2017	L	L	S	S	S	S	S	L	L	S
Peng et al., 2017	L	L	S	S	H	S	S	L	L	S
Liu et al., 2020	L	L	S	S	S	S	S	L	L	S
Raun et al., 2022	S	L	S	S	S	S	S	L	L	L
Shih et al., 2017	L	L	S	S	S	S	S	L	L	S
Wang et al., 2022	S	L	S	S	S	S	S	L	L	L
Wen et al., 2011	S	L	S	S	S	S	S	L	L	S
Xu et al., 2021	S	L	S	S	S	S	S	L	L	S
Yang et al., 2022	S	L	S	S	S	S	S	L	L	S

H, high risk of bias; L, low risk of bias; S, some risk of bias (Unclear).

Table S5a. Inconsistency test outcomes for ALT

Comparison	No.Studies	NMA	Direct	Indirect	Difference	95CI_L	95CI_U	p-Value
Antroqunonol_High_Dose:Antroqunonol_Low_Dose	2	-3.55	-3.55	NA	NA	NA	NA	NA
Antroqunonol_High_Dose:Antroqunonol_Medium_Dose	2	-4.48	-4.49	NA	NA	NA	NA	NA
Antroqunonol_High_Dose:Model_Control	2	-24.13	9.01	-136.03	145.04	107.04	183.04	0.00
Antroqunonol_High_Dose:Negative_Control	2	27.05	-10.57	143.04	-153.61	-190.94	-116.28	0.00
Antroqunonol_High_Dose:Polysaccharid_High_Dose	0	6.98	NA	6.98	NA	NA	NA	NA
Antroqunonol_High_Dose:Polysaccharid_Low_Dose	0	1.43	NA	1.43	NA	NA	NA	NA
Antroqunonol_High_Dose:Polysaccharid_Medium_Dose	0	2.20	NA	2.20	NA	NA	NA	NA
Antroqunonol_High_Dose:Positive_Control	2	-1.16	0.66	-9.88	10.54	-33.31	54.39	0.64
Antroqunonol_High_Dose:Triterpenodis_High_Dose	0	18.24	NA	18.24	NA	NA	NA	NA
Antroqunonol_High_Dose:Triterpenodis_Low_Dose	0	-8.26	NA	-8.26	NA	NA	NA	NA
Antroqunonol_High_Dose:Triterpenodis_Medium_Dose	0	5.75	NA	5.75	NA	NA	NA	NA
Antroqunonol_Low_Dose:Antroqunonol_Medium_Dose	2	-0.94	-0.92	NA	NA	NA	NA	NA
Antroqunonol_Low_Dose:Model_Control	2	-20.58	12.52	-133.10	145.62	107.42	183.83	0.00
Antroqunonol_Low_Dose:Negative_Control	2	30.59	-7.03	147.55	-154.58	-192.12	-117.05	0.00
Antroqunonol_Low_Dose:Polysaccharid_High_Dose	0	10.53	NA	10.53	NA	NA	NA	NA
Antroqunonol_Low_Dose:Polysaccharid_Low_Dose	0	4.97	NA	4.97	NA	NA	NA	NA
Antroqunonol_Low_Dose:Polysaccharid_Medium_Dose	0	5.75	NA	5.75	NA	NA	NA	NA
Antroqunonol_Low_Dose:Positive_Control	2	2.38	4.21	-6.40	10.60	-33.48	54.69	0.64
Antroqunonol_Low_Dose:Triterpenodis_High_Dose	0	21.78	NA	21.78	NA	NA	NA	NA
Antroqunonol_Low_Dose:Triterpenodis_Low_Dose	0	-4.71	NA	-4.71	NA	NA	NA	NA
Antroqunonol_Low_Dose:Triterpenodis_Medium_Dose	0	9.29	NA	9.29	NA	NA	NA	NA
Antroqunonol_Medium_Dose:Model_Control	2	-19.64	13.50	-131.79	145.29	107.22	183.36	0.00

Antroqunonol_Medium_Dose:Negative_Control	2	31.53	-6.17	148.08	-154.25	-191.65	-116.86	0.00
Antroqunonol_Medium_Dose:Polysaccharid_High_Dose	0	11.47	NA	11.47	NA	NA	NA	NA
Antroqunonol_Medium_Dose:Polysaccharid_Low_Dose	0	5.91	NA	5.91	NA	NA	NA	NA
Antroqunonol_Medium_Dose:Polysaccharid_Medium_Dose	0	6.69	NA	6.69	NA	NA	NA	NA
Antroqunonol_Medium_Dose:Positive_Control	2	3.32	5.14	-5.40	10.54	-33.38	54.46	0.64
Antroqunonol_Medium_Dose:Triterpenodis_High_Dose	0	22.72	NA	22.72	NA	NA	NA	NA
Antroqunonol_Medium_Dose:Triterpenodis_Low_Dose	0	-3.77	NA	-3.77	NA	NA	NA	NA
Antroqunonol_Medium_Dose:Triterpenodis_Medium_Dose	0	10.23	NA	10.23	NA	NA	NA	NA
Negative_Control:Model_Control	10	-51.18	-54.85	-35.98	-18.87	-41.72	3.97	0.11
Polysaccharid_High_Dose:Model_Control	6	-31.11	-52.16	-3.61	-48.55	-73.30	-23.79	0.00
Polysaccharid_Low_Dose:Model_Control	6	-25.56	-36.07	-11.17	-24.90	-49.38	-0.42	0.05
Polysaccharid_Medium_Dose:Model_Control	3	-26.33	-7.25	-40.34	33.09	-1.49	67.68	0.06
Positive_Control:Model_Control	6	-22.97	-2.33	-117.22	114.89	84.19	145.59	0.00
Triterpenodis_High_Dose:Model_Control	5	-42.37	-49.23	-12.24	-36.99	-67.40	-6.58	0.02
Triterpenodis_Low_Dose:Model_Control	4	-15.87	-13.57	-24.56	10.99	-20.87	42.86	0.50
Triterpenodis_Medium_Dose:Model_Control	3	-29.87	-16.67	-74.90	58.23	25.67	90.79	0.00
Negative_Control:Polysaccharid_High_Dose	5	-20.06	-27.77	-11.06	-16.71	-40.25	6.82	0.16
Negative_Control:Polysaccharid_Low_Dose	5	-25.62	-40.97	-7.48	-33.49	-56.72	-10.25	0.00
Negative_Control:Polysaccharid_Medium_Dose	3	-24.84	-7.07	-37.40	30.34	-3.85	64.52	0.08
Negative_Control:Positive_Control	5	-28.21	2.78	-83.02	85.80	60.92	110.68	0.00
Negative_Control:Triterpenodis_High_Dose	5	-8.81	-11.34	8.26	-19.60	-53.73	14.52	0.26
Negative_Control:Triterpenodis_Low_Dose	4	-35.31	-34.96	-36.87	1.91	-31.25	35.06	0.91
Negative_Control:Triterpenodis_Medium_Dose	3	-21.30	-10.00	-61.04	51.04	18.60	83.49	0.00
Polysaccharid_High_Dose:Polysaccharid_Low_Dose	7	-5.56	-9.21	26.31	-35.52	-74.02	2.99	0.07

Polysaccharid_High_Dose:Polysaccharid_Medium_Dose	4	-4.78	0.04	-23.87	23.90	-17.26	65.07	0.26
Polysaccharid_High_Dose:Positive_Control	3	-8.15	-13.40	-5.84	-7.55	-39.35	24.24	0.64
Polysaccharid_High_Dose:Triterpenodis_High_Dose	0	11.26	NA	11.26	NA	NA	NA	NA
Polysaccharid_High_Dose:Triterpenodis_Low_Dose	0	-15.24	NA	-15.24	NA	NA	NA	NA
Polysaccharid_High_Dose:Triterpenodis_Medium_Dose	0	-1.24	NA	-1.24	NA	NA	NA	NA
Polysaccharid_Low_Dose:Polysaccharid_Medium_Dose	4	0.78	3.10	-8.50	11.60	-29.50	52.70	0.58
Polysaccharid_Low_Dose:Positive_Control	3	-2.59	-1.40	-3.11	1.72	-29.79	33.22	0.92
Polysaccharid_Low_Dose:Triterpenodis_High_Dose	0	16.81	NA	16.81	NA	NA	NA	NA
Polysaccharid_Low_Dose:Triterpenodis_Low_Dose	0	-9.69	NA	-9.69	NA	NA	NA	NA
Polysaccharid_Low_Dose:Triterpenodis_Medium_Dose	0	4.32	NA	4.32	NA	NA	NA	NA
Polysaccharid_Medium_Dose:Positive_Control	2	-3.37	NA	-4.36	NA	NA	NA	NA
Polysaccharid_Medium_Dose:Triterpenodis_High_Dose	0	16.04	NA	16.04	NA	NA	NA	NA
Polysaccharid_Medium_Dose:Triterpenodis_Low_Dose	0	-10.46	NA	-10.46	NA	NA	NA	NA
Polysaccharid_Medium_Dose:Triterpenodis_Medium_Dose	0	3.54	NA	3.54	NA	NA	NA	NA
Positive_Control:Triterpenodis_High_Dose	1	19.40	-0.43	28.91	-29.34	-60.52	1.85	0.07
Positive_Control:Triterpenodis_Low_Dose	1	-7.10	-0.81	-10.66	9.84	-22.27	41.96	0.55
Positive_Control:Triterpenodis_Medium_Dose	1	6.91	-0.40	11.53	-11.93	-44.68	20.82	0.48
Triterpenodis_High_Dose:Triterpenodis_Low_Dose	4	-26.50	-21.03	-95.77	74.73	22.55	126.92	0.01
Triterpenodis_High_Dose:Triterpenodis_Medium_Dose	3	-12.49	-5.62	-59.57	53.95	10.96	96.93	0.01
Triterpenodis_Low_Dose:Triterpenodis_Medium_Dose	3	14.00	10.08	83.19	-73.11	-138.83	-7.39	0.03

95CI-L: lower limit of 95% confidence interval; 95CI-U: upper limit of 95% confidence interval; NMA: network meta-analysis.

Table S5b. Inconsistency test outcomes for AST

Comparison	No.Studies	NMA	Direct	Indirect	Difference	95CI_L	95CI_U	p-Value
Antroqunonol_High_Dose:Antroqunonol_Low_Dose	2	0.04	0.04	NA	NA	NA	NA	NA
Antroqunonol_High_Dose:Antroqunonol_Medium_Dose	2	-2.16	-2.16	NA	NA	NA	NA	NA
Antroqunonol_High_Dose:Model_Control	2	-28.82	6.44	-146.87	153.31	88.46	218.16	0.00
Antroqunonol_High_Dose:Negative_Control	2	19.82	-21.38	141.99	-163.37	-225.99	-100.76	0.00
Antroqunonol_High_Dose:Polysaccharid_High_Dose	0	12.10	NA	12.10	NA	NA	NA	NA
Antroqunonol_High_Dose:Polysaccharid_Low_Dose	0	3.75	NA	3.75	NA	NA	NA	NA
Antroqunonol_High_Dose:Polysaccharid_Medium_Dose	0	-1.29	NA	-1.29	NA	NA	NA	NA
Antroqunonol_High_Dose:Positive_Control	2	0.71	4.51	-17.91	22.42	-53.21	98.06	0.56
Antroqunonol_High_Dose:Triterpenodis_High_Dose	0	8.14	NA	8.14	NA	NA	NA	NA
Antroqunonol_High_Dose:Triterpenodis_Low_Dose	0	-6.93	NA	-6.93	NA	NA	NA	NA
Antroqunonol_High_Dose:Triterpenodis_Medium_Dose	0	21.36	NA	21.36	NA	NA	NA	NA
Antroqunonol_Low_Dose:Antroqunonol_Medium_Dose	2	-2.20	-2.20	NA	NA	NA	NA	NA
Antroqunonol_Low_Dose:Model_Control	2	-28.86	6.39	-146.89	153.28	88.43	218.14	0.00
Antroqunonol_Low_Dose:Negative_Control	2	19.78	-21.40	141.92	-163.32	-225.94	-100.70	0.00
Antroqunonol_Low_Dose:Polysaccharid_High_Dose	0	12.06	NA	12.06	NA	NA	NA	NA
Antroqunonol_Low_Dose:Polysaccharid_Low_Dose	0	3.71	NA	3.71	NA	NA	NA	NA
Antroqunonol_Low_Dose:Polysaccharid_Medium_Dose	0	-1.33	NA	-1.33	NA	NA	NA	NA
Antroqunonol_Low_Dose:Positive_Control	2	0.68	4.47	-17.92	22.39	-53.25	98.02	0.56
Antroqunonol_Low_Dose:Triterpenodis_High_Dose	0	8.10	NA	8.10	NA	NA	NA	NA
Antroqunonol_Low_Dose:Triterpenodis_Low_Dose	0	-6.97	NA	-6.97	NA	NA	NA	NA
Antroqunonol_Low_Dose:Triterpenodis_Medium_Dose	0	21.32	NA	21.32	NA	NA	NA	NA

Antroqunonol_Medium_Dose:Model_Control	2	-26.67	8.63	-144.53	153.16	88.44	217.88	0.00
Antroqunonol_Medium_Dose:Negative_Control	2	21.97	-19.29	143.99	-163.28	-225.77	-100.79	0.00
Antroqunonol_Medium_Dose:Polysaccharid_High_Dose	0	14.25	NA	14.25	NA	NA	NA	NA
Antroqunonol_Medium_Dose:Polysaccharid_Low_Dose	0	5.91	NA	5.91	NA	NA	NA	NA
Antroqunonol_Medium_Dose:Polysaccharid_Medium_Dose	0	0.87	NA	0.87	NA	NA	NA	NA
Antroqunonol_Medium_Dose:Positive_Control	2	2.87	6.69	-15.81	22.50	-52.99	97.98	0.56
Antroqunonol_Medium_Dose:Triterpenodis_High_Dose	0	10.30	NA	10.30	NA	NA	NA	NA
Antroqunonol_Medium_Dose:Triterpenodis_Low_Dose	0	-4.78	NA	-4.78	NA	NA	NA	NA
Antroqunonol_Medium_Dose:Triterpenodis_Medium_Dose	0	23.51	NA	23.51	NA	NA	NA	NA
Negative_Control:Model_Control	10	-48.64	-47.17	-54.55	7.38	-28.84	43.61	0.69
Polysaccharid_High_Dose:Model_Control	6	-40.92	-58.73	-9.62	-49.11	-87.96	-10.27	0.01
Polysaccharid_Low_Dose:Model_Control	6	-32.58	-42.65	-12.47	-30.18	-70.77	10.41	0.15
Polysaccharid_Medium_Dose:Model_Control	3	-27.53	-15.37	-36.05	20.67	-36.08	77.42	0.48
Positive_Control:Model_Control	5	-29.54	-3.03	-79.03	76.01	33.14	118.87	0.00
Triterpenodis_High_Dose:Model_Control	5	-36.96	-43.37	-3.51	-39.86	-92.65	12.93	0.14
Triterpenodis_Low_Dose:Model_Control	4	-21.89	-19.54	-31.31	11.77	-42.64	66.17	0.67
Triterpenodis_Medium_Dose:Model_Control	3	-50.18	-40.81	-75.94	35.13	-17.18	87.44	0.19
Negative_Control:Polysaccharid_High_Dose	5	-7.72	-18.61	5.97	-24.59	-62.52	13.34	0.20
Negative_Control:Polysaccharid_Low_Dose	5	-16.06	-23.33	-6.80	-16.53	-55.22	22.17	0.40
Negative_Control:Polysaccharid_Medium_Dose	3	-21.11	-26.92	-16.92	-10.00	-66.67	46.66	0.73
Negative_Control:Positive_Control	6	-19.10	2.76	-110.69	113.45	63.24	163.67	0.00
Negative_Control:Triterpenodis_High_Dose	5	-11.68	-14.82	8.63	-23.45	-79.07	32.17	0.41
Negative_Control:Triterpenodis_Low_Dose	4	-26.75	-31.84	-2.66	-29.18	-85.41	27.05	0.31
Negative_Control:Triterpenodis_Medium_Dose	3	1.54	1.91	0.30	1.61	-52.39	55.61	0.95

Polysaccharid_High_Dose:Polysaccharid_Low_Dose	7	-8.35	-11.65	20.81	-32.46	-94.95	30.02	0.31
Polysaccharid_High_Dose:Polysaccharid_Medium_Dose	4	-13.39	-3.36	-49.78	46.43	-19.86	112.71	0.17
Polysaccharid_High_Dose:Positive_Control	3	-11.38	-25.66	-5.06	-20.60	-72.81	31.62	0.44
Polysaccharid_High_Dose:Triterpenodis_High_Dose	0	-3.96	NA	-3.96	NA	NA	NA	NA
Polysaccharid_High_Dose:Triterpenodis_Low_Dose	0	-19.03	NA	-19.03	NA	NA	NA	NA
Polysaccharid_High_Dose:Triterpenodis_Medium_Dose	0	9.26	NA	9.26	NA	NA	NA	NA
Polysaccharid_Low_Dose:Polysaccharid_Medium_Dose	4	-5.04	3.76	-37.41	41.17	-25.62	107.97	0.23
Polysaccharid_Low_Dose:Positive_Control	2	-3.04	NA	-4.25	NA	NA	NA	NA
Polysaccharid_Low_Dose:Triterpenodis_High_Dose	0	4.39	NA	4.39	NA	NA	NA	NA
Polysaccharid_Low_Dose:Triterpenodis_Low_Dose	0	-10.68	NA	-10.68	NA	NA	NA	NA
Polysaccharid_Low_Dose:Triterpenodis_Medium_Dose	0	17.60	NA	17.60	NA	NA	NA	NA
Polysaccharid_Medium_Dose:Positive_Control	2	2.00	NA	-0.08	NA	NA	NA	NA
Polysaccharid_Medium_Dose:Triterpenodis_High_Dose	0	9.43	NA	9.43	NA	NA	NA	NA
Polysaccharid_Medium_Dose:Triterpenodis_Low_Dose	0	-5.64	NA	-5.64	NA	NA	NA	NA
Polysaccharid_Medium_Dose:Triterpenodis_Medium_Dose	0	22.64	NA	22.64	NA	NA	NA	NA
Positive_Control:Triterpenodis_High_Dose	1	7.43	-2.86	12.05	-14.91	-68.15	38.32	0.58
Positive_Control:Triterpenodis_Low_Dose	1	-7.65	3.56	-13.82	17.38	-37.50	72.27	0.53
Positive_Control:Triterpenodis_Medium_Dose	1	20.64	0.10	33.41	-33.31	-89.42	22.81	0.24
Triterpenodis_High_Dose:Triterpenodis_Low_Dose	4	-15.07	-9.44	-82.11	72.68	-13.42	158.77	0.10
Triterpenodis_High_Dose:Triterpenodis_Medium_Dose	3	13.22	21.32	-37.48	58.81	-11.46	129.08	0.10
Triterpenodis_Low_Dose:Triterpenodis_Medium_Dose	3	28.29	23.82	65.01	-41.19	-123.54	41.16	0.33

95CI-L: lower limit of 95% confidence interval; 95CI-U: upper limit of 95% confidence interval; NMA: network meta-analysis.

Table S5c. Inconsistency test outcomes for MDA

Comparison	No.Studies	NMA	Direct	Indirect	Difference	95CI_L	95CI_U	p-Value
Antroqunonol_High_Dose:Antroqunonol_Low_Dose	2	-0.53	-0.53	NA	NA	NA	NA	NA
Antroqunonol_High_Dose:Antroqunonol_Medium_Dose	2	-0.25	-0.25	NA	NA	NA	NA	NA
Antroqunonol_High_Dose:Model_Control	2	-8.83	-0.37	-35.93	35.56	21.95	49.17	0.00
Antroqunonol_High_Dose:Negative_Control	2	7.58	-0.23	31.67	-31.89	-45.31	-18.48	0.00
Antroqunonol_High_Dose:Polysaccharid_High_Dose	0	-0.64	NA	-0.64	NA	NA	NA	NA
Antroqunonol_High_Dose:Polysaccharid_Low_Dose	0	-1.34	NA	-1.34	NA	NA	NA	NA
Antroqunonol_High_Dose:Polysaccharid_Medium_Dose	0	-0.70	NA	-0.70	NA	NA	NA	NA
Antroqunonol_High_Dose:Positive_Control	2	0.32	-0.33	2.86	-3.19	-17.86	11.47	0.67
Antroqunonol_High_Dose:Triterpenodis_High_Dose	0	10.22	NA	10.22	NA	NA	NA	NA
Antroqunonol_High_Dose:Triterpenodis_Low_Dose	0	-6.54	NA	-6.54	NA	NA	NA	NA
Antroqunonol_High_Dose:Triterpenodis_Medium_Dose	0	2.34	NA	2.34	NA	NA	NA	NA
Antroqunonol_Low_Dose:Antroqunonol_Medium_Dose	2	0.29	0.29	NA	NA	NA	NA	NA
Antroqunonol_Low_Dose:Model_Control	2	-8.30	0.16	-35.40	35.56	21.95	49.17	0.00
Antroqunonol_Low_Dose:Negative_Control	2	8.11	0.30	32.20	-31.89	-45.31	-18.48	0.00
Antroqunonol_Low_Dose:Polysaccharid_High_Dose	0	-0.11	NA	-0.11	NA	NA	NA	NA
Antroqunonol_Low_Dose:Polysaccharid_Low_Dose	0	-0.81	NA	-0.81	NA	NA	NA	NA
Antroqunonol_Low_Dose:Polysaccharid_Medium_Dose	0	-0.17	NA	-0.17	NA	NA	NA	NA
Antroqunonol_Low_Dose:Positive_Control	2	0.85	0.20	3.39	-3.19	-17.86	11.47	0.67
Antroqunonol_Low_Dose:Triterpenodis_High_Dose	0	10.75	NA	10.75	NA	NA	NA	NA
Antroqunonol_Low_Dose:Triterpenodis_Low_Dose	0	-6.01	NA	-6.01	NA	NA	NA	NA
Antroqunonol_Low_Dose:Triterpenodis_Medium_Dose	0	2.87	NA	2.87	NA	NA	NA	NA

Antroqunonol_Medium_Dose:Model_Control	2	-8.58	-0.13	-35.68	35.56	21.95	49.17	0.00
Antroqunonol_Medium_Dose:Negative_Control	2	7.83	0.02	31.91	-31.89	-45.31	-18.48	0.00
Antroqunonol_Medium_Dose:Polysaccharid_High_Dose	0	-0.39	NA	-0.39	NA	NA	NA	NA
Antroqunonol_Medium_Dose:Polysaccharid_Low_Dose	0	-1.09	NA	-1.09	NA	NA	NA	NA
Antroqunonol_Medium_Dose:Polysaccharid_Medium_Dose	0	-0.46	NA	-0.46	NA	NA	NA	NA
Antroqunonol_Medium_Dose:Positive_Control	2	0.56	-0.09	3.10	-3.19	-17.86	11.47	0.67
Antroqunonol_Medium_Dose:Triterpenodis_High_Dose	0	10.47	NA	10.47	NA	NA	NA	NA
Antroqunonol_Medium_Dose:Triterpenodis_Low_Dose	0	-6.29	NA	-6.29	NA	NA	NA	NA
Antroqunonol_Medium_Dose:Triterpenodis_Medium_Dose	0	2.58	NA	2.58	NA	NA	NA	NA
Negative_Control:Model_Control	7	-16.41	-19.57	-0.52	-19.05	-27.78	-10.33	0.00
Polysaccharid_High_Dose:Model_Control	4	-8.19	-0.40	-24.82	24.42	16.11	32.73	0.00
Polysaccharid_Low_Dose:Model_Control	4	-7.49	-0.28	-30.20	29.92	20.36	39.49	0.00
Polysaccharid_Medium_Dose:Model_Control	2	-8.13	-0.19	-18.20	18.00	8.00	28.00	0.00
Positive_Control:Model_Control	4	-9.15	-0.15	-32.17	32.03	23.17	40.88	0.00
Triterpenodis_High_Dose:Model_Control	3	-19.05	-33.83	45.57	-79.40	-92.13	-66.67	0.00
Triterpenodis_Low_Dose:Model_Control	3	-2.29	-17.39	64.45	-81.83	-94.70	-68.97	0.00
Triterpenodis_Medium_Dose:Model_Control	2	-11.16	-3.77	-31.98	28.21	15.03	41.38	0.00
Negative_Control:Polysaccharid_High_Dose	4	-8.22	-0.20	-26.20	26.00	17.55	34.45	0.00
Negative_Control:Polysaccharid_Low_Dose	3	-8.92	-0.38	-21.97	21.59	12.98	30.21	0.00
Negative_Control:Polysaccharid_Medium_Dose	2	-8.28	-0.05	-19.03	18.98	8.90	29.06	0.00
Negative_Control:Positive_Control	5	-7.26	-0.29	-46.05	45.76	35.01	56.51	0.00
Negative_Control:Triterpenodis_High_Dose	3	2.64	-11.92	67.04	-78.96	-91.67	-66.24	0.00
Negative_Control:Triterpenodis_Low_Dose	3	-14.12	-28.66	50.75	-79.42	-92.26	-66.58	0.00
Negative_Control:Triterpenodis_Medium_Dose	2	-5.24	-0.75	-18.36	17.61	4.38	30.83	0.01

Polysaccharid_High_Dose:Polysaccharid_Low_Dose	5	-0.70	-0.09	-11.73	11.64	-6.73	30.00	0.21
Polysaccharid_High_Dose:Polysaccharid_Medium_Dose	3	-0.06	-0.03	-0.23	0.21	-12.74	13.16	0.97
Polysaccharid_High_Dose:Positive_Control	2	0.96	-0.32	2.10	-2.42	-11.56	6.71	0.60
Polysaccharid_High_Dose:Triterpenodis_High_Dose	0	10.86	NA	10.86	NA	NA	NA	NA
Polysaccharid_High_Dose:Triterpenodis_Low_Dose	0	-5.90	NA	-5.90	NA	NA	NA	NA
Polysaccharid_High_Dose:Triterpenodis_Medium_Dose	0	2.98	NA	2.98	NA	NA	NA	NA
Polysaccharid_Low_Dose:Polysaccharid_Medium_Dose	3	0.64	0.08	4.02	-3.94	-18.35	10.47	0.59
Polysaccharid_Low_Dose:Positive_Control	1	1.66	-0.10	2.32	-2.42	-13.43	8.59	0.67
Polysaccharid_Low_Dose:Triterpenodis_High_Dose	0	11.56	NA	11.56	NA	NA	NA	NA
Polysaccharid_Low_Dose:Triterpenodis_Low_Dose	0	-5.20	NA	-5.20	NA	NA	NA	NA
Polysaccharid_Low_Dose:Triterpenodis_Medium_Dose	0	3.68	NA	3.68	NA	NA	NA	NA
Polysaccharid_Medium_Dose:Positive_Control	1	1.02	-0.30	1.73	-2.03	-13.68	9.61	0.73
Polysaccharid_Medium_Dose:Triterpenodis_High_Dose	0	10.92	NA	10.92	NA	NA	NA	NA
Polysaccharid_Medium_Dose:Triterpenodis_Low_Dose	0	-5.84	NA	-5.84	NA	NA	NA	NA
Polysaccharid_Medium_Dose:Triterpenodis_Medium_Dose	0	3.04	NA	3.04	NA	NA	NA	NA
Positive_Control:Triterpenodis_High_Dose	1	9.90	0.12	15.28	-15.16	-26.84	-3.48	0.01
Positive_Control:Triterpenodis_Low_Dose	1	-6.86	-0.27	-10.53	10.26	-1.45	21.98	0.09
Positive_Control:Triterpenodis_Medium_Dose	1	2.02	0.06	3.60	-3.54	-16.16	9.08	0.58
Triterpenodis_High_Dose:Triterpenodis_Low_Dose	3	-16.76	-16.81	NA	NA	NA	NA	NA
Triterpenodis_High_Dose:Triterpenodis_Medium_Dose	2	-7.88	0.98	-66.68	67.65	48.99	86.31	0.00
Triterpenodis_Low_Dose:Triterpenodis_Medium_Dose	2	8.88	2.90	46.45	-43.55	-61.96	-25.15	0.00

95CI-L: lower limit of 95% confidence interval; 95CI-U: upper limit of 95% confidence interval; NMA: network meta-analysis.

Table S5d. Inconsistency test outcomes for TNF-α

Comparison	Studies	NMA	Direct	Indirect	Difference	95CI_L	95CI_U	p-Value
Negative_Control:Model_Control	5	-87.44	-87.92	NA	NA	NA	NA	NA
Polysaccharid_High_Dose:Model_Control	2	-46.73	-12.12	-244.01	231.89	129.50	334.27	0.00
Polysaccharid_Low_Dose:Model_Control	2	-42.31	-7.69	-239.65	231.96	129.54	334.38	0.00
Positive_Control:Model_Control	2	-60.41	-122.60	203.66	-326.26	-419.65	-232.87	0.00
Triterpenodis_High_Dose:Model_Control	3	-88.75	-113.54	128.68	-242.23	-344.26	-140.19	0.00
Triterpenodis_Low_Dose:Model_Control	3	-49.36	-69.62	132.66	-202.28	-306.66	-97.90	0.00
Triterpenodis_Medium_Dose:Model_Control	2	-89.27	-151.92	170.17	-322.09	-413.66	-230.52	0.00
Negative_Control:Polysaccharid_High_Dose	2	-40.71	-6.20	-238.55	232.35	129.79	334.91	0.00
Negative_Control:Polysaccharid_Low_Dose	2	-45.13	-10.62	-243.06	232.44	129.84	335.03	0.00
Negative_Control:Positive_Control	2	-27.03	-72.22	169.09	-241.31	-335.20	-147.43	0.00
Negative_Control:Triterpenodis_High_Dose	3	1.30	-21.88	208.66	-230.54	-333.21	-127.87	0.00
Negative_Control:Triterpenodis_Low_Dose	3	-38.08	-58.96	150.76	-209.71	-314.21	-105.21	0.00
Negative_Control:Triterpenodis_Medium_Dose	2	1.82	-43.92	195.73	-239.65	-331.73	-147.56	0.00
Polysaccharid_High_Dose:Polysaccharid_Low_Dose	2	-4.42	-4.42	NA	NA	NA	NA	NA
Polysaccharid_High_Dose:Positive_Control	0	13.68	NA	13.68	NA	NA	NA	NA
Polysaccharid_High_Dose:Triterpenodis_High_Dose	0	42.01	NA	42.01	NA	NA	NA	NA
Polysaccharid_High_Dose:Triterpenodis_Low_Dose	0	2.63	NA	2.63	NA	NA	NA	NA
Polysaccharid_High_Dose:Triterpenodis_Medium_Dose	0	42.53	NA	42.53	NA	NA	NA	NA
Polysaccharid_Low_Dose:Positive_Control	0	18.10	NA	18.10	NA	NA	NA	NA
Polysaccharid_Low_Dose:Triterpenodis_High_Dose	0	46.44	NA	46.44	NA	NA	NA	NA
Polysaccharid_Low_Dose:Triterpenodis_Low_Dose	0	7.05	NA	7.05	NA	NA	NA	NA
Polysaccharid_Low_Dose:Triterpenodis_Medium_Dose	0	46.96	NA	46.96	NA	NA	NA	NA

Positive_Control:Triterpenodis_High_Dose	2	28.34	43.95	-82.78	126.73	10.91	242.55	0.03
Positive_Control:Triterpenodis_Low_Dose	2	-11.05	-13.90	7.07	-20.97	-133.30	91.35	0.71
Positive_Control:Triterpenodis_Medium_Dose	2	28.86	28.68	NA	NA	NA	NA	NA
Triterpenodis_High_Dose:Triterpenodis_Low_Dose	3	-39.38	-38.16	-987.76	949.61	32.59	1866.63	0.04
Triterpenodis_High_Dose:Triterpenodis_Medium_Dose	2	0.52	-15.45	111.71	-127.16	-240.77	-13.56	0.03
Triterpenodis_Low_Dose:Triterpenodis_Medium_Dose	2	39.90	41.71	28.68	13.03	-97.05	123.11	0.82
Polysaccharid_Medium_Dose:Triterpenodis_Low_Dose	0	-68.78	NA	-68.78	NA	NA	NA	NA
Polysaccharid_Medium_Dose:Triterpenodis_Medium_Dose	0	-58.73	NA	-58.73	NA	NA	NA	NA
Positive_Control:Triterpenodis_High_Dose	1	-5.36	6.10	-20.60	26.70	-61.81	115.22	0.55
Positive_Control:Triterpenodis_Low_Dose	1	-11.05	-1.10	-24.50	23.40	-64.87	111.67	0.60
Positive_Control:Triterpenodis_Medium_Dose	1	-1.00	7.60	-37.66	45.26	-88.48	179.00	0.51
Triterpenodis_High_Dose:Triterpenodis_Low_Dose	2	-5.69	-5.72	NA	NA	NA	NA	NA
Triterpenodis_High_Dose:Triterpenodis_Medium_Dose	1	4.36	1.50	16.33	-14.83	-146.18	116.52	0.82
Triterpenodis_Low_Dose:Triterpenodis_Medium_Dose	1	10.05	8.70	15.96	-7.26	-139.99	125.47	0.91

95CI-L: lower limit of 95% confidence interval; 95CI-U: upper limit of 95% confidence interval; NMA: network meta-analysis.

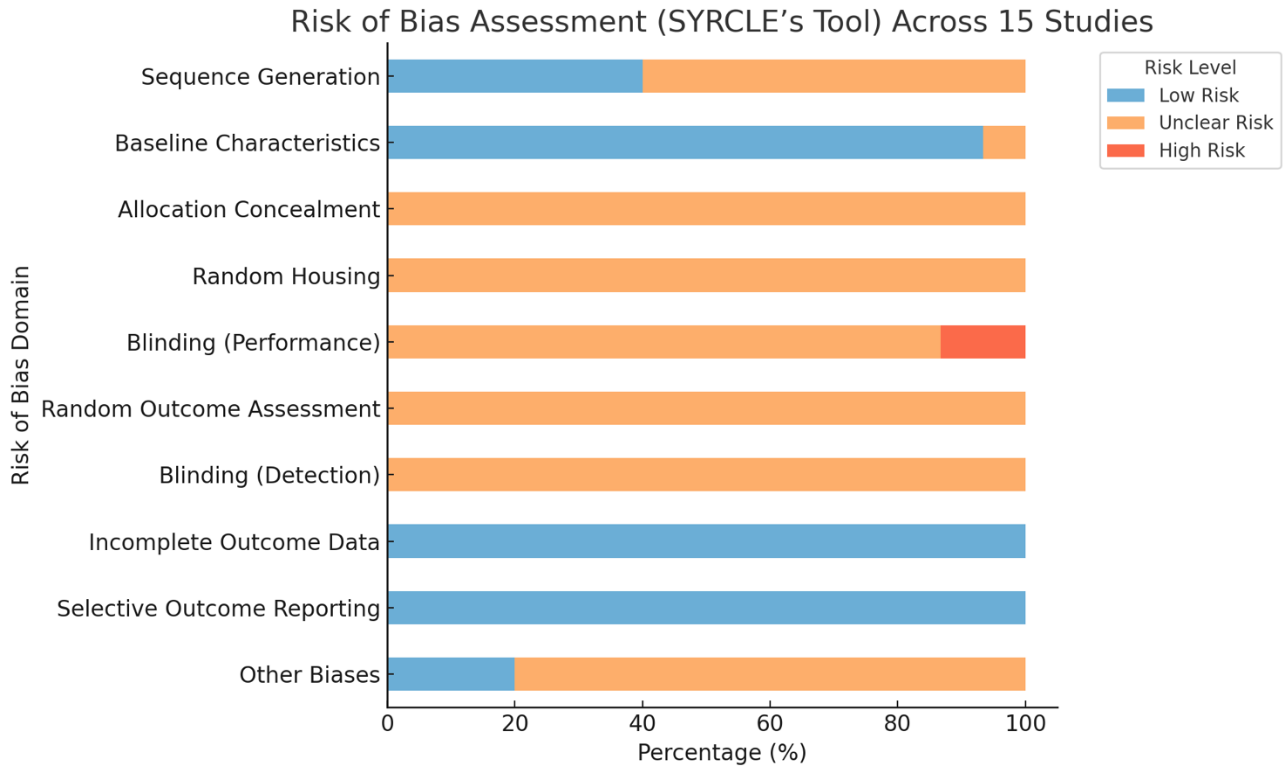

Figure S1. The figure illustrates the distribution of risk of bias assessments across 15 studies using the SYRCLE's Risk of Bias tool.

Figure S2a_ALT Individual study results (with selected studies excluded) grouped by treatment comparison

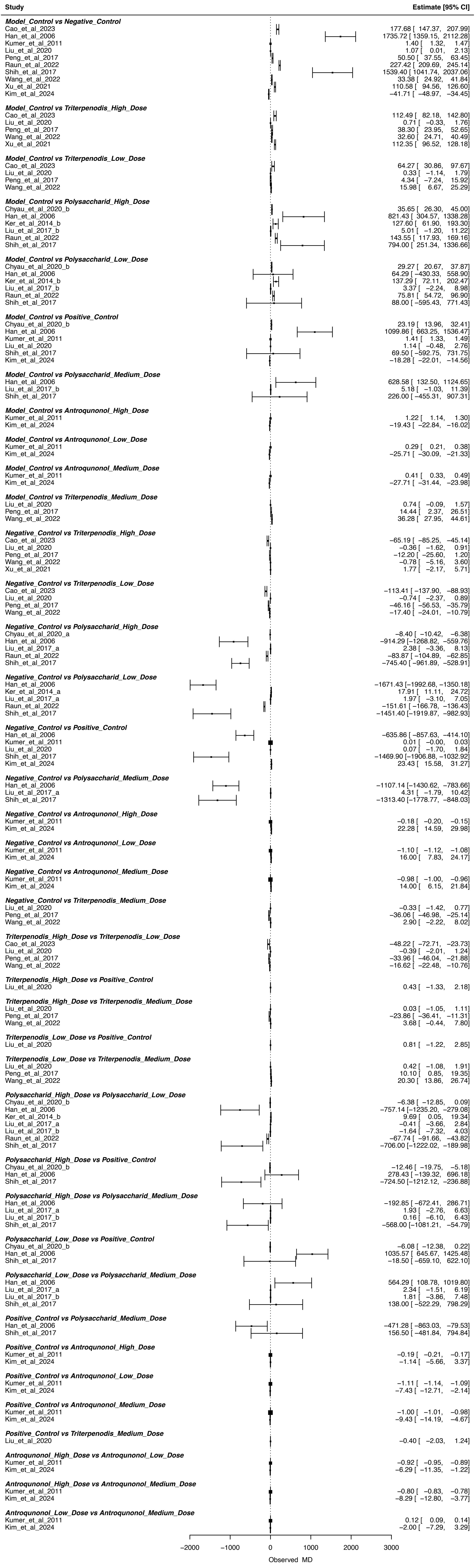

Figure S2b_AST Individual study results (with selected studies excluded) grouped by treatment comparison

Study	Estimate [95% CI]
Model_Control vs Negative_Control	
Cao_et_al_2023	84.05 [66.02, 102.08]
Han_et_al_2006	1783.29 [1449.48, 2117.10]
Kim_et_al_2024	−60.83 [−71.75, −49.91]
Kumer_et_al_2011	3.19 [3.11, 3.27]
Liu_et_al_2020	8.46 [3.18, 13.74]
Peng_et_al_2017	50.50 [37.55, 63.45]
Raun_et_al_2022	115.36 [105.20, 125.52]
Shih_et_al_2017	1653.20 [1030.04, 2276.36]
Wang_et_al_2022	80.62 [52.15, 109.09]
Xu_et_al_2021	72.95 [61.21, 84.69]
Model_Control vs Triterpenodis_High_Dose	
Cao_et_al_2023	46.98 [21.53, 72.43]
Liu_et_al_2020	2.55 [−2.49, 7.59]
Peng_et_al_2017	38.30 [23.95, 52.65]
Wang_et_al_2022	38.42 [9.17, 67.67]
Xu_et_al_2021	92.36 [80.40, 104.32]
Model_Control vs Triterpenodis_Low_Dose	
Cao_et_al_2023	37.49 [13.52, 61.46]
Liu_et_al_2020	8.97 [5.35, 12.59]
Peng_et_al_2017	4.34 [−7.24, 15.92]
Wang_et_al_2022	40.20 [−1.05, 81.45]
Model_Control vs Polysaccharid_High_Dose	
Chyau_et_al_2020_b	65.41 [59.31, 71.51]
Han_et_al_2006	779.15 [367.32, 1190.98]
Ker_et_al_2014_b	60.56 [22.33, 98.79]
Liu_et_al_2017_b	11.11 [7.15, 15.07]
Raun_et_al_2022	88.44 [76.18, 100.70]
Shih_et_al_2017	1012.50 [349.37, 1675.63]
Model_Control vs Positive_Control	
Han_et_al_2006	941.47 [449.40, 1433.54]
Kim_et_al_2024	−7.22 [−13.11, −1.33]
Kumer_et_al_2011	3.21 [3.10, 3.32]
Liu_et_al_2020	5.41 [−0.87, 11.69]
Shih_et_al_2017	78.00 [−739.88, 895.88]
Model_Control vs Polysaccharid_Low_Dose	
Chyau_et_al_2020_b	50.41 [44.98, 55.84]
Han_et_al_2006	158.30 [−409.01, 725.61]
Ker_et_al_2014_b	58.70 [17.54, 99.86]
Liu_et_al_2017_b	8.70 [4.41, 12.99]
Raun_et_al_2022	59.87 [49.96, 69.78]
Shih_et_al_2017	187.50 [−612.91, 987.91]
Model_Control vs Polysaccharid_Medium_Dose	
Han_et_al_2006	424.96 [14.78, 835.14]
Liu_et_al_2017_b	10.00 [5.94, 14.06]
Shih_et_al_2017	267.50 [−536.81, 1071.81]
Model_Control vs Antroqunonol_Low_Dose	
Kim_et_al_2024	−14.95 [−22.68, −7.21]
Kumer_et_al_2011	1.90 [1.77, 2.03]
Model_Control vs Antroqunonol_Medium_Dose	
Kim_et_al_2024	−19.59 [−26.77, −12.41]
Kumer_et_al_2011	2.04 [1.79, 2.29]
Model_Control vs Antroqunonol_High_Dose	
Kim_et_al_2024	−15.98 [−23.71, −8.25]
Kumer_et_al_2011	2.81 [2.72, 2.90]
Model_Control vs Triterpenodis_Medium_Dose	
Liu_et_al_2020	5.51 [1.89, 9.13]
Peng_et_al_2017	14.44 [2.37, 26.51]
Wang_et_al_2022	127.30 [97.95, 156.65]
Negative_Control vs Triterpenodis_High_Dose	
Cao_et_al_2023	−37.07 [−55.87, −18.27]
Liu_et_al_2020	−5.91 [−12.01, 0.19]
Peng_et_al_2017	−12.20 [−25.60, 1.20]
Wang_et_al_2022	−42.20 [−56.10, −28.30]
Xu_et_al_2021	19.41 [9.35, 29.47]
Negative_Control vs Triterpenodis_Low_Dose	
Cao_et_al_2023	−46.56 [−63.31, −29.81]
Liu_et_al_2020	0.51 [−4.49, 5.51]
Peng_et_al_2017	−46.16 [−56.53, −35.79]
Wang_et_al_2022	−40.42 [−72.65, −8.19]
Negative_Control vs Polysaccharid_High_Dose	
Chyau_et_al_2020_a	5.82 [1.72, 9.92]
Han_et_al_2006	−1004.14 [−1302.33, −705.95]
Liu_et_al_2017_a	8.15 [4.22, 12.08]
Raun_et_al_2022	−26.92 [−37.08, −16.76]
Shih_et_al_2017	−640.70 [−867.52, −413.88]
Negative_Control vs Positive_Control	
Chyau_et_al_2020_a	−16.25 [−20.49, −12.01]
Han_et_al_2006	−841.82 [−1243.63, −440.01]
Kim_et_al_2024	53.61 [42.45, 64.77]
Kumer_et_al_2011	0.02 [−0.09, 0.13]
Liu_et_al_2020	−3.05 [−10.21, 4.11]
Shih_et_al_2017	−1575.20 [−2104.96, −1045.44]
Negative_Control vs Polysaccharid_Low_Dose	
Han_et_al_2006	−1624.99 [−2116.06, −1133.92]
Ker_et_al_2014_a	3.12 [−10.30, 16.54]
Liu_et_al_2017_a	7.41 [3.77, 11.05]
Raun_et_al_2022	−55.49 [−62.64, −48.34]
Shih_et_al_2017	−1465.70 [−1968.06, −963.34]
Negative_Control vs Polysaccharid_Medium_Dose	
Han_et_al_2006	−1358.33 [−1654.24, −1062.42]
Liu_et_al_2017_a	11.67 [7.81, 15.53]
Shih_et_al_2017	−1385.70 [−1894.26, −877.14]
Negative_Control vs Antroqunonol_Low_Dose	
Kim_et_al_2024	45.88 [33.65, 58.11]
Kumer_et_al_2011	−1.29 [−1.42, −1.16]
Negative_Control vs Antroqunonol_Medium_Dose	
Kim_et_al_2024	41.24 [29.35, 53.13]
Kumer_et_al_2011	−1.15 [−1.40, −0.90]
Negative_Control vs Antroqunonol_High_Dose	
Kim_et_al_2024	44.85 [32.62, 57.08]
Kumer_et_al_2011	−0.38 [−0.48, −0.28]
Negative_Control vs Triterpenodis_Medium_Dose	
Liu_et_al_2020	−2.95 [−7.95, 2.05]
Peng_et_al_2017	−36.06 [−46.98, −25.14]
Wang_et_al_2022	46.68 [32.57, 60.79]
Triterpenodis_High_Dose vs Triterpenodis_Low_Dose	
Cao_et_al_2023	−9.49 [−34.05, 15.07]
Liu_et_al_2020	6.42 [1.67, 11.17]
Peng_et_al_2017	−33.96 [−46.04, −21.88]
Wang_et_al_2022	1.78 [−31.14, 34.70]
Triterpenodis_High_Dose vs Positive_Control	
Liu_et_al_2020	2.86 [−4.13, 9.85]
Triterpenodis_High_Dose vs Triterpenodis_Medium_Dose	
Liu_et_al_2020	2.96 [−1.79, 7.71]
Peng_et_al_2017	−23.86 [−36.41, −11.31]
Wang_et_al_2022	88.88 [73.26, 104.50]
Triterpenodis_Low_Dose vs Positive_Control	
Liu_et_al_2020	−3.56 [−9.61, 2.49]
Triterpenodis_Low_Dose vs Triterpenodis_Medium_Dose	
Liu_et_al_2020	−3.46 [−6.66, −0.26]
Peng_et_al_2017	10.10 [0.85, 19.35]
Wang_et_al_2022	87.10 [54.09, 120.11]
Polysaccharid_High_Dose vs Positive_Control	
Chyau_et_al_2020_a	−22.07 [−25.55, −18.59]
Han_et_al_2006	162.32 [−306.32, 630.96]
Shih_et_al_2017	−934.50 [−1510.74, −358.26]
Polysaccharid_High_Dose vs Polysaccharid_Low_Dose	
Chyau_et_al_2020_b	−15.00 [−19.30, −10.70]
Han_et_al_2006	−620.85 [−1167.96, −73.74]
Ker_et_al_2014_b	−1.86 [−20.44, 16.72]
Liu_et_al_2017_a	−0.74 [−4.46, 2.98]
Liu_et_al_2017_b	−2.41 [−5.48, 0.66]
Raun_et_al_2022	−28.57 [−38.48, −18.66]
Shih_et_al_2017	−825.00 [−1376.16, −273.84]
Polysaccharid_High_Dose vs Polysaccharid_Medium_Dose	
Han_et_al_2006	−354.19 [−735.95, 27.57]
Liu_et_al_2017_a	3.52 [−0.41, 7.45]
Liu_et_al_2017_b	−1.11 [−3.85, 1.63]
Shih_et_al_2017	−745.00 [−1301.82, −188.18]
Positive_Control vs Polysaccharid_Low_Dose	
Han_et_al_2006	−783.17 [−1392.97, −173.37]
Shih_et_al_2017	109.50 [−620.55, 839.55]
Positive_Control vs Polysaccharid_Medium_Dose	
Han_et_al_2006	−516.51 [−983.70, −49.32]
Shih_et_al_2017	189.50 [−544.83, 923.83]
Positive_Control vs Antroqunonol_Low_Dose	
Kim_et_al_2024	−7.73 [−15.80, 0.34]
Kumer_et_al_2011	−1.31 [−1.46, −1.16]
Positive_Control vs Antroqunonol_Medium_Dose	
Kim_et_al_2024	−12.37 [−19.91, −4.83]
Kumer_et_al_2011	−1.17 [−1.43, −0.91]
Positive_Control vs Antroqunonol_High_Dose	
Kim_et_al_2024	−8.76 [−16.83, −0.70]
Kumer_et_al_2011	−0.40 [−0.52, −0.28]
Positive_Control vs Triterpenodis_Medium_Dose	
Liu_et_al_2020	0.10 [−5.95, 6.15]
Polysaccharid_Low_Dose vs Polysaccharid_Medium_Dose	
Han_et_al_2006	266.66 [−279.21, 812.53]
Liu_et_al_2017_a	4.26 [0.62, 7.90]
Liu_et_al_2017_b	1.30 [−1.90, 4.50]
Shih_et_al_2017	80.00 [−634.82, 794.82]
Antroqunonol_Low_Dose vs Antroqunonol_Medium_Dose	
Kim_et_al_2024	−4.64 [−13.69, 4.41]
Kumer_et_al_2011	0.14 [−0.13, 0.41]
Antroqunonol_Low_Dose vs Antroqunonol_High_Dose	
Kim_et_al_2024	−1.03 [−10.53, 8.46]
Kumer_et_al_2011	0.91 [0.77, 1.05]
Antroqunonol_Medium_Dose vs Antroqunonol_High_Dose	
Kim_et_al_2024	3.61 [−5.44, 12.66]
Kumer_et_al_2011	0.77 [0.51, 1.03]

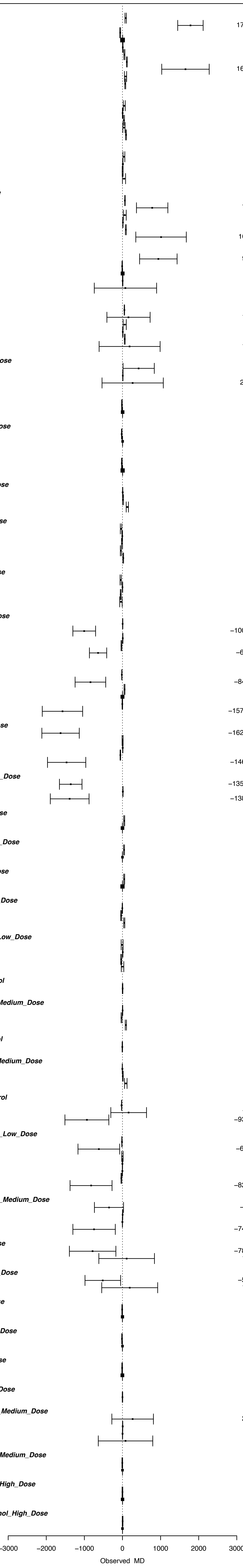

Figure S3a_MDA Individual study results (with selected studies excluded) grouped by treatment comparison

Study	Estimate [95% CI]
Model_Control vs Negative_Control	
Cao_et_al_2023	125.32 [125.08, 125.56]
Kim_et_al_2024	−0.88 [−0.97, −0.79]
Kumer_et_al_2011	1.17 [1.07, 1.27]
Shih_et_al_2017	0.50 [0.22, 0.78]
Wang_et_al_2022	8.60 [6.72, 10.48]
Wen_et_al_2011	0.68 [0.62, 0.74]
Yang_et_al_2022	1.24 [1.17, 1.31]
Model_Control vs Triterpenodis_High_Dose	
Cao_et_al_2023	93.50 [93.25, 93.75]
Wang_et_al_2022	5.10 [2.34, 7.86]
Wen_et_al_2011	0.63 [0.56, 0.70]
Model_Control vs Triterpenodis_Low_Dose	
Cao_et_al_2023	48.70 [48.42, 48.98]
Wang_et_al_2022	1.42 [−1.95, 4.79]
Wen_et_al_2011	0.24 [0.17, 0.31]
Model_Control vs Polysaccharid_High_Dose	
Chyau_et_al_2020_b	0.33 [0.30, 0.36]
Liu_et_al_2017_b	0.10 [0.07, 0.13]
Shih_et_al_2017	0.40 [0.14, 0.66]
Yang_et_al_2022	0.76 [0.66, 0.86]
Model_Control vs Positive_Control	
Kim_et_al_2024	−0.73 [−0.79, −0.67]
Kumer_et_al_2011	0.80 [0.75, 0.85]
Shih_et_al_2017	0.00 [−0.31, 0.31]
Wen_et_al_2011	0.51 [0.44, 0.58]
Model_Control vs Polysaccharid_Low_Dose	
Chyau_et_al_2020_b	0.51 [0.47, 0.55]
Liu_et_al_2017_b	0.08 [0.05, 0.11]
Shih_et_al_2017	0.10 [−0.25, 0.45]
Yang_et_al_2022	0.44 [0.34, 0.54]
Model_Control vs Antroqunonol_Low_Dose	
Kim_et_al_2024	−0.37 [−0.42, −0.32]
Kumer_et_al_2011	0.05 [0.01, 0.09]
Model_Control vs Antroqunonol_Medium_Dose	
Kim_et_al_2024	−0.32 [−0.38, −0.26]
Kumer_et_al_2011	0.57 [0.55, 0.59]
Model_Control vs Antroqunonol_High_Dose	
Kim_et_al_2024	−0.25 [−0.30, −0.20]
Kumer_et_al_2011	0.99 [0.96, 1.02]
Model_Control vs Polysaccharid_Medium_Dose	
Liu_et_al_2017_b	0.09 [0.06, 0.12]
Shih_et_al_2017	0.30 [0.04, 0.56]
Model_Control vs Triterpenodis_Medium_Dose	
Wang_et_al_2022	7.19 [4.78, 9.60]
Wen_et_al_2011	0.57 [0.49, 0.65]
Negative_Control vs Triterpenodis_High_Dose	
Cao_et_al_2023	−31.82 [−32.01, −31.63]
Wang_et_al_2022	−3.50 [−5.56, −1.44]
Wen_et_al_2011	−0.05 [−0.11, 0.01]
Negative_Control vs Triterpenodis_Low_Dose	
Cao_et_al_2023	−76.62 [−76.84, −76.40]
Wang_et_al_2022	−7.18 [−10.00, −4.36]
Wen_et_al_2011	−0.44 [−0.49, −0.39]
Negative_Control vs Polysaccharid_High_Dose	
Chyau_et_al_2020_a	−0.30 [−0.32, −0.28]
Liu_et_al_2017_a	0.07 [0.06, 0.08]
Shih_et_al_2017	−0.10 [−0.24, 0.04]
Yang_et_al_2022	−0.48 [−0.55, −0.41]
Negative_Control vs Positive_Control	
Chyau_et_al_2020_a	−0.54 [−0.57, −0.51]
Kim_et_al_2024	0.15 [0.05, 0.25]
Kumer_et_al_2011	−0.37 [−0.48, −0.26]
Shih_et_al_2017	−0.50 [−0.72, −0.28]
Wen_et_al_2011	−0.17 [−0.22, −0.12]
Negative_Control vs Polysaccharid_Low_Dose	
Liu_et_al_2017_a	0.07 [0.05, 0.09]
Shih_et_al_2017	−0.40 [−0.68, −0.12]
Yang_et_al_2022	−0.80 [−0.87, −0.73]
Negative_Control vs Antroqunonol_Low_Dose	
Kim_et_al_2024	0.51 [0.41, 0.61]
Kumer_et_al_2011	−1.12 [−1.22, −1.02]
Negative_Control vs Antroqunonol_Medium_Dose	
Kim_et_al_2024	0.56 [0.46, 0.66]
Kumer_et_al_2011	−0.60 [−0.70, −0.50]
Negative_Control vs Antroqunonol_High_Dose	
Kim_et_al_2024	0.63 [0.53, 0.73]
Kumer_et_al_2011	−0.18 [−0.28, −0.08]
Negative_Control vs Polysaccharid_Medium_Dose	
Liu_et_al_2017_a	0.10 [0.08, 0.12]
Shih_et_al_2017	−0.20 [−0.34, −0.06]
Negative_Control vs Triterpenodis_Medium_Dose	
Wang_et_al_2022	−1.41 [−2.96, 0.14]
Wen_et_al_2011	−0.11 [−0.17, −0.05]
Triterpenodis_High_Dose vs Triterpenodis_Low_Dose	
Cao_et_al_2023	−44.80 [−45.04, −44.56]
Wang_et_al_2022	−3.68 [−7.15, −0.21]
Wen_et_al_2011	−0.39 [−0.45, −0.33]
Triterpenodis_High_Dose vs Positive_Control	
Wen_et_al_2011	−0.12 [−0.18, −0.06]
Triterpenodis_High_Dose vs Triterpenodis_Medium_Dose	
Wang_et_al_2022	2.09 [−0.46, 4.64]
Wen_et_al_2011	−0.06 [−0.13, 0.01]
Triterpenodis_Low_Dose vs Positive_Control	
Wen_et_al_2011	0.27 [0.21, 0.33]
Triterpenodis_Low_Dose vs Triterpenodis_Medium_Dose	
Wang_et_al_2022	5.77 [2.57, 8.97]
Wen_et_al_2011	0.33 [0.26, 0.40]
Polysaccharid_High_Dose vs Positive_Control	
Chyau_et_al_2020_a	−0.24 [−0.27, −0.21]
Shih_et_al_2017	−0.40 [−0.60, −0.20]
Polysaccharid_High_Dose vs Polysaccharid_Low_Dose	
Chyau_et_al_2020_b	0.18 [0.14, 0.22]
Liu_et_al_2017_a	0.00 [−0.02, 0.02]
Liu_et_al_2017_b	−0.02 [−0.04, 0.00]
Shih_et_al_2017	−0.30 [−0.56, −0.04]
Yang_et_al_2022	−0.32 [−0.42, −0.22]
Polysaccharid_High_Dose vs Polysaccharid_Medium_Dose	
Liu_et_al_2017_a	0.03 [0.02, 0.04]
Liu_et_al_2017_b	−0.01 [−0.03, 0.01]
Shih_et_al_2017	−0.10 [−0.19, −0.01]
Positive_Control vs Polysaccharid_Low_Dose	
Shih_et_al_2017	0.10 [−0.21, 0.41]
Positive_Control vs Antroqunonol_Low_Dose	
Kim_et_al_2024	0.36 [0.29, 0.43]
Kumer_et_al_2011	−0.75 [−0.81, −0.69]
Positive_Control vs Antroqunonol_Medium_Dose	
Kim_et_al_2024	0.41 [0.34, 0.48]
Kumer_et_al_2011	−0.23 [−0.28, −0.18]
Positive_Control vs Antroqunonol_High_Dose	
Kim_et_al_2024	0.48 [0.41, 0.55]
Kumer_et_al_2011	0.19 [0.14, 0.24]
Positive_Control vs Polysaccharid_Medium_Dose	
Shih_et_al_2017	0.30 [0.10, 0.50]
Positive_Control vs Triterpenodis_Medium_Dose	
Wen_et_al_2011	0.06 [−0.01, 0.13]
Polysaccharid_Low_Dose vs Polysaccharid_Medium_Dose	
Liu_et_al_2017_a	0.03 [0.01, 0.05]
Liu_et_al_2017_b	0.01 [−0.01, 0.03]
Shih_et_al_2017	0.20 [−0.06, 0.46]
Antroqunonol_Low_Dose vs Antroqunonol_Medium_Dose	
Kim_et_al_2024	0.05 [−0.02, 0.12]
Kumer_et_al_2011	0.52 [0.48, 0.56]
Antroqunonol_Low_Dose vs Antroqunonol_High_Dose	
Kim_et_al_2024	0.12 [0.06, 0.18]
Kumer_et_al_2011	0.94 [0.90, 0.98]
Antroqunonol_Medium_Dose vs Antroqunonol_High_Dose	
Kim_et_al_2024	0.07 [0.00, 0.14]
Kumer_et_al_2011	0.42 [0.39, 0.45]

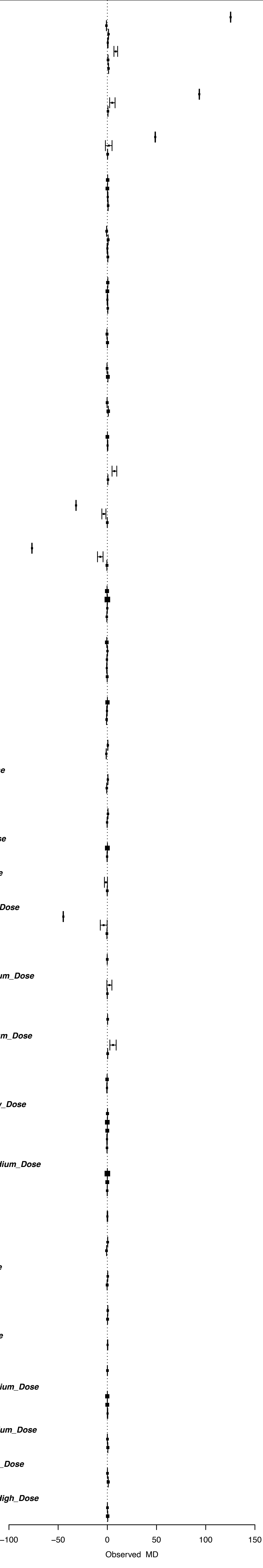

Figure S3b_TNF-α Individual study results (with selected studies excluded) grouped by treatment comparison

Study	Estimate [95% CI]
Model_Control vs Negative_Control	
Cao_et_al_2023	19.55 [16.78, 22.32]
Liu_et_al_2020	12.30 [2.32, 22.28]
Raun_et_al_2022	31.18 [27.76, 34.60]
Wen_et_al_2011	377.77 [367.53, 388.01]
Yang_et_al_2022	5.49 [5.22, 5.76]
Model_Control vs Triterpenodis_High_Dose	
Cao_et_al_2023	10.36 [7.30, 13.42]
Liu_et_al_2020	13.10 [3.30, 22.90]
Wen_et_al_2011	321.42 [310.57, 332.27]
Model_Control vs Triterpenodis_Low_Dose	
Cao_et_al_2023	6.07 [2.57, 9.57]
Liu_et_al_2020	5.90 [−0.96, 12.76]
Wen_et_al_2011	212.70 [192.18, 233.22]
Model_Control vs Positive_Control	
Liu_et_al_2020	7.00 [−6.01, 20.01]
Wen_et_al_2011	240.47 [225.17, 255.77]
Model_Control vs Triterpenodis_Medium_Dose	
Liu_et_al_2020	14.60 [3.06, 26.14]
Wen_et_al_2011	289.68 [277.70, 301.66]
Model_Control vs Polysaccharid_High_Dose	
Raun_et_al_2022	20.39 [16.86, 23.93]
Yang_et_al_2022	3.87 [3.60, 4.14]
Model_Control vs Polysaccharid_Low_Dose	
Raun_et_al_2022	13.68 [9.87, 17.50]
Yang_et_al_2022	1.73 [1.44, 2.02]
Negative_Control vs Triterpenodis_High_Dose	
Cao_et_al_2023	−9.19 [−11.19, −7.19]
Liu_et_al_2020	0.80 [−11.50, 13.10]
Wen_et_al_2011	−56.35 [−60.37, −52.33]
Negative_Control vs Triterpenodis_Low_Dose	
Cao_et_al_2023	−13.48 [−16.11, −10.85]
Liu_et_al_2020	−6.40 [−16.52, 3.72]
Wen_et_al_2011	−165.07 [−182.94, −147.20]
Negative_Control vs Positive_Control	
Liu_et_al_2020	−5.30 [−20.28, 9.68]
Wen_et_al_2011	−137.30 [−148.80, −125.80]
Negative_Control vs Triterpenodis_Medium_Dose	
Liu_et_al_2020	2.30 [−11.43, 16.03]
Wen_et_al_2011	−88.09 [−94.57, −81.61]
Negative_Control vs Polysaccharid_High_Dose	
Raun_et_al_2022	−10.79 [−12.14, −9.43]
Yang_et_al_2022	−1.62 [−1.75, −1.49]
Negative_Control vs Polysaccharid_Low_Dose	
Raun_et_al_2022	−17.50 [−19.47, −15.52]
Yang_et_al_2022	−3.76 [−3.92, −3.60]
Triterpenodis_High_Dose vs Triterpenodis_Low_Dose	
Cao_et_al_2023	−4.29 [−7.22, −1.36]
Liu_et_al_2020	−7.20 [−17.14, 2.74]
Wen_et_al_2011	−108.72 [−126.95, −90.49]
Triterpenodis_High_Dose vs Positive_Control	
Liu_et_al_2020	−6.10 [−20.96, 8.76]
Wen_et_al_2011	−80.95 [−93.00, −68.90]
Triterpenodis_High_Dose vs Triterpenodis_Medium_Dose	
Liu_et_al_2020	1.50 [−12.09, 15.09]
Wen_et_al_2011	−31.74 [−39.15, −24.33]
Triterpenodis_Low_Dose vs Positive_Control	
Liu_et_al_2020	1.10 [−12.01, 14.21]
Wen_et_al_2011	27.77 [6.59, 48.95]
Triterpenodis_Low_Dose vs Triterpenodis_Medium_Dose	
Liu_et_al_2020	8.70 [−2.95, 20.35]
Wen_et_al_2011	76.98 [58.05, 95.91]
Positive_Control vs Triterpenodis_Medium_Dose	
Liu_et_al_2020	7.60 [−8.46, 23.66]
Wen_et_al_2011	49.21 [36.13, 62.29]
Polysaccharid_High_Dose vs Polysaccharid_Low_Dose	
Raun_et_al_2022	−6.71 [−8.89, −4.53]
Yang_et_al_2022	−2.14 [−2.31, −1.97]

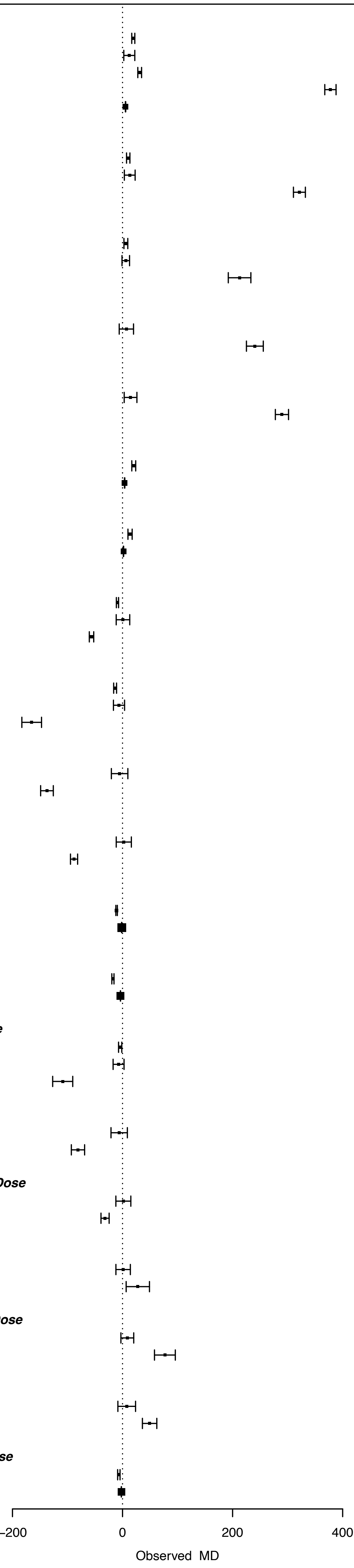

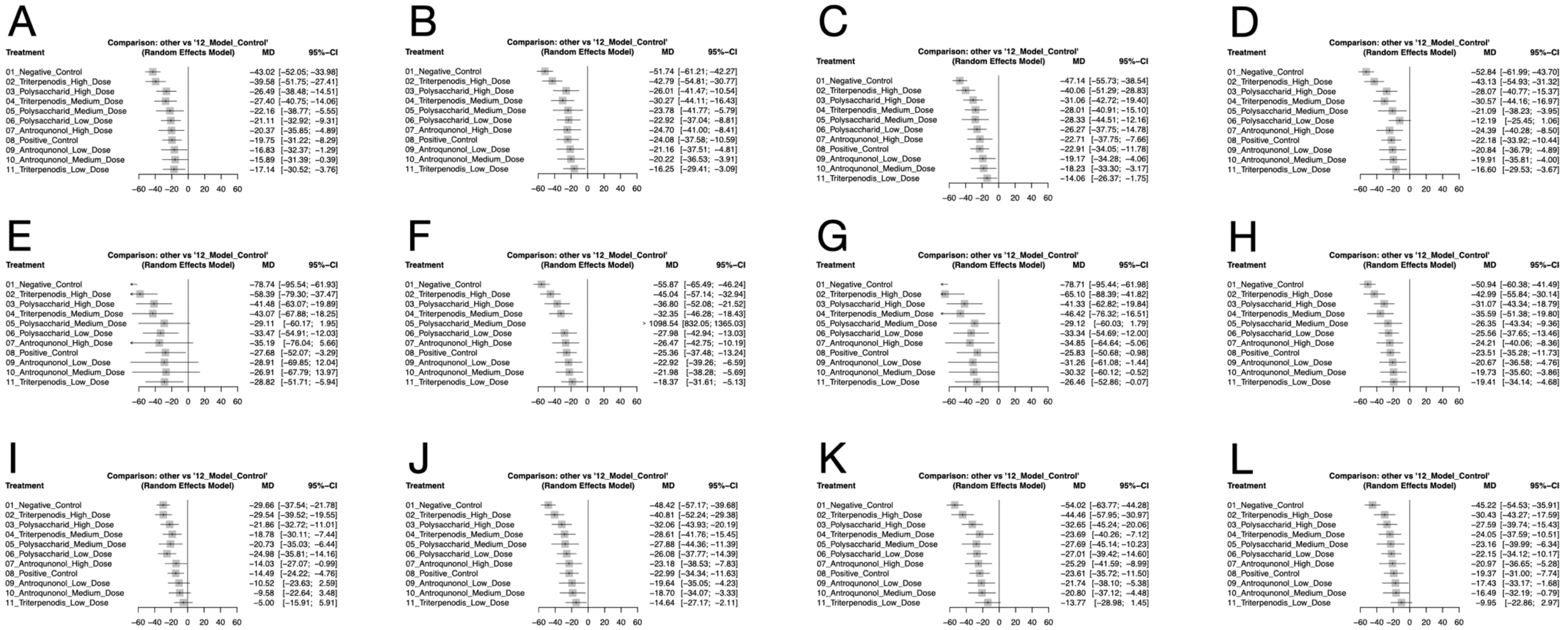

Studies Excluded One by One (Referenced Alphabetically)

a. Cao et al., 2023
b. Chyau et al., 2020
c. Han et al., 2006
d. Ker et al., 2014
e. Kumer et al., 2011
f. Liu et al., 2017
g. Liu et al., 2020
h. Peng et al., 2017
i. Raun et al., 2022
j. Shih et al., 2017
k. Wang et al., 2022
l. Xu et al., 2021

*NA / Kim et al., 2024

Fig. S4A The forest plots display the results of the sensitivity analysis conducted using the one-study removal method, involving 12 studies (labeled a to l).

ALT

Fig-S4a

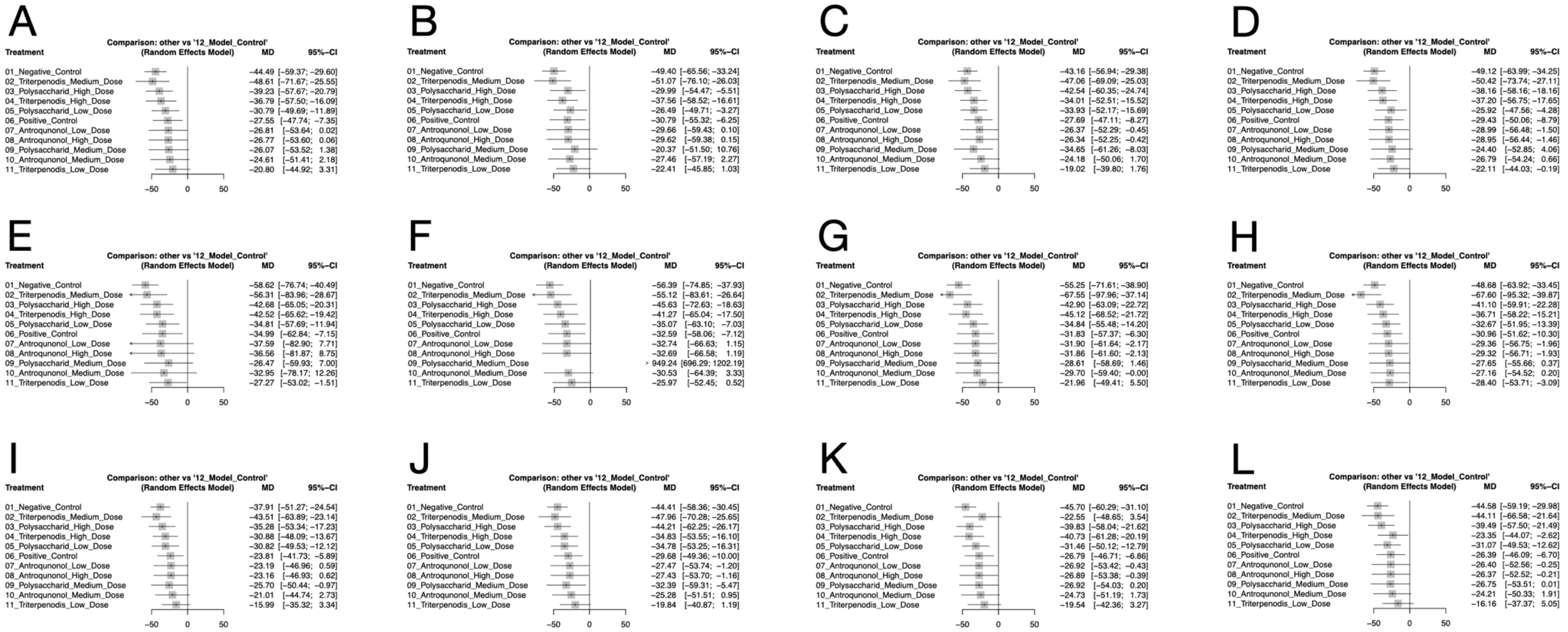

Studies Excluded One by One (Referenced Alphabetically)

a. Cao et al., 2023
b. Chyau et al., 2020
c. Han et al., 2006
d. Ker et al., 2014
e. Kumer et al., 2011
f. Liu et al., 2017
g. Liu et al., 2020
h. Peng et al., 2017
i. Raun et al., 2022
j. Shih et al., 2017
k. Wang et al., 2022
l. Xu et al., 2021

*NA / Kim et al., 2024

Fig. S4A The forest plots display the results of the sensitivity analysis conducted using the one-study removal method, involving 12 studies (labeled a to l).

AST

Fig-S4b

Antrodia cinnamomea on Liver Function Biomarkers

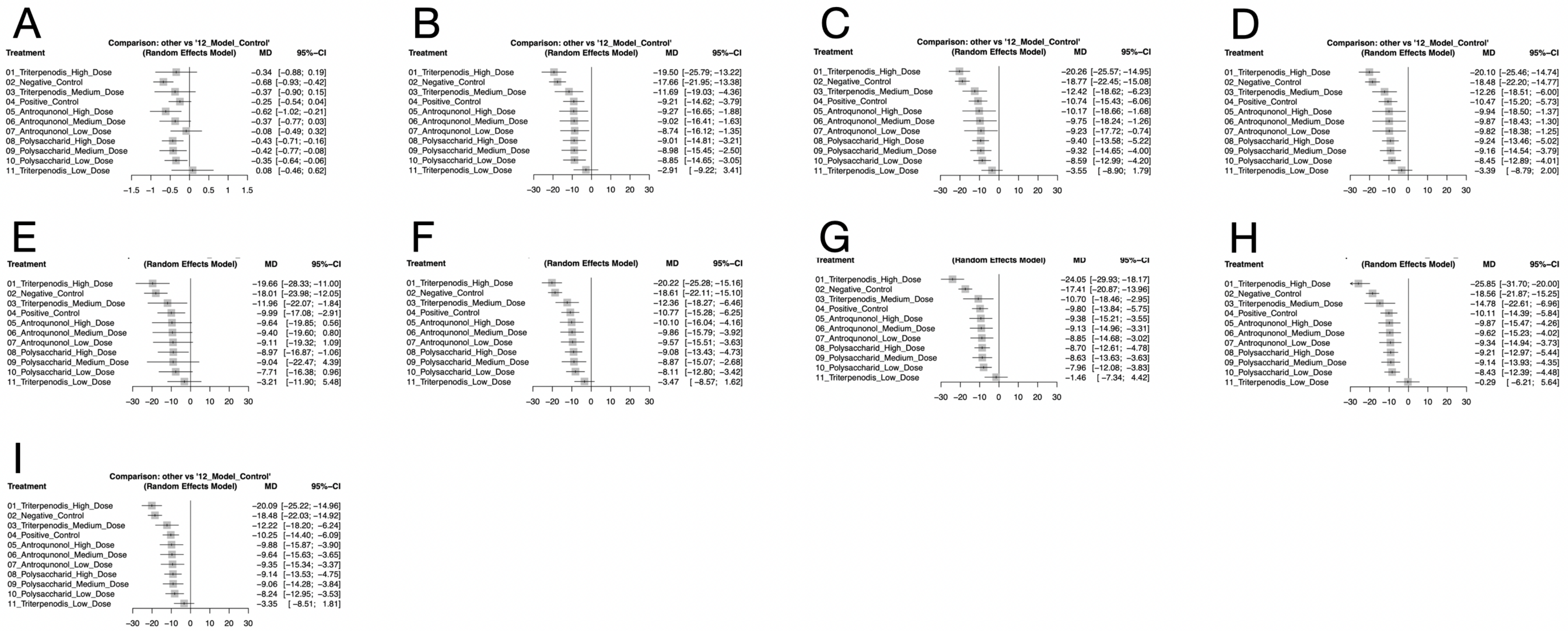

Studies Excluded One by One (Referenced Alphabetically)

a. Cao et al., 2023
b. Chyau et al., 2020
c. Kim et al., 2024
d. Kumer et al., 2011
e. Liu et al., 2017
f. Shih et al., 2017
g. Wang et al., 2022
h. Wen et al., 2011
i. Yang et al., 2022

Fig. S4C The forest plots display the results of the sensitivity analysis conducted using the one-study removal method, involving 9 studies (labeled a to i).

MDA Fig-S4c

Antrodia cinnamomea on Liver Function Biomarkers

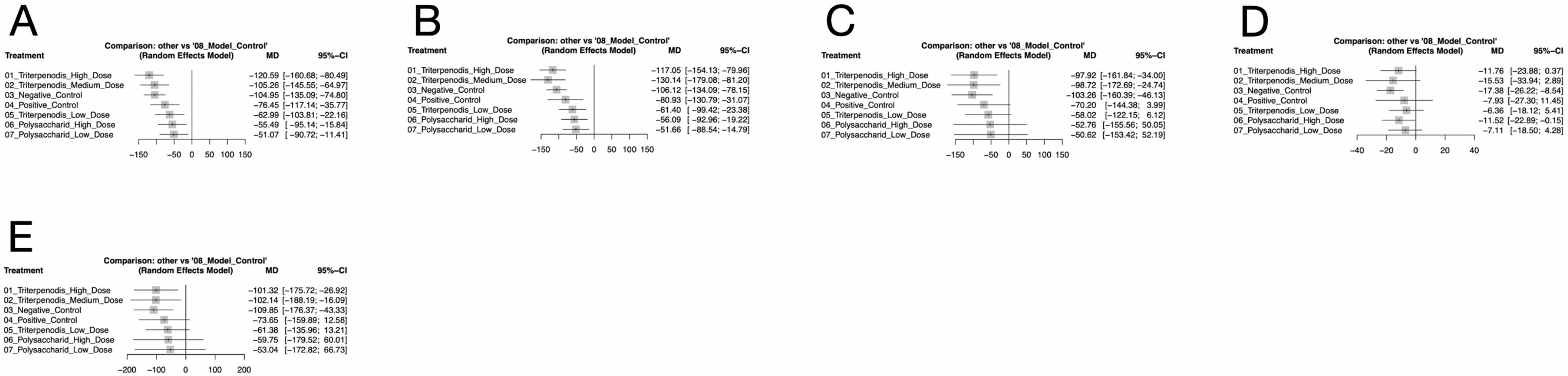

Studies Excluded One by One (Referenced Alphabetically)

a. Cao et al., 2023
b. Liu et al., 2020
c. Raun et al., 2022
d. Wen et al., 2011
e. Yang et al., 2022

Fig. S4D The forest plots display the results of the sensitivity analysis conducted using the one-study removal method, involving 5 studies (labeled a to e).

TNF-α Fig-S4d

Antrodia cinnamomea on Liver Function Biomarkers

Figure S5a: Publication bias of ALT

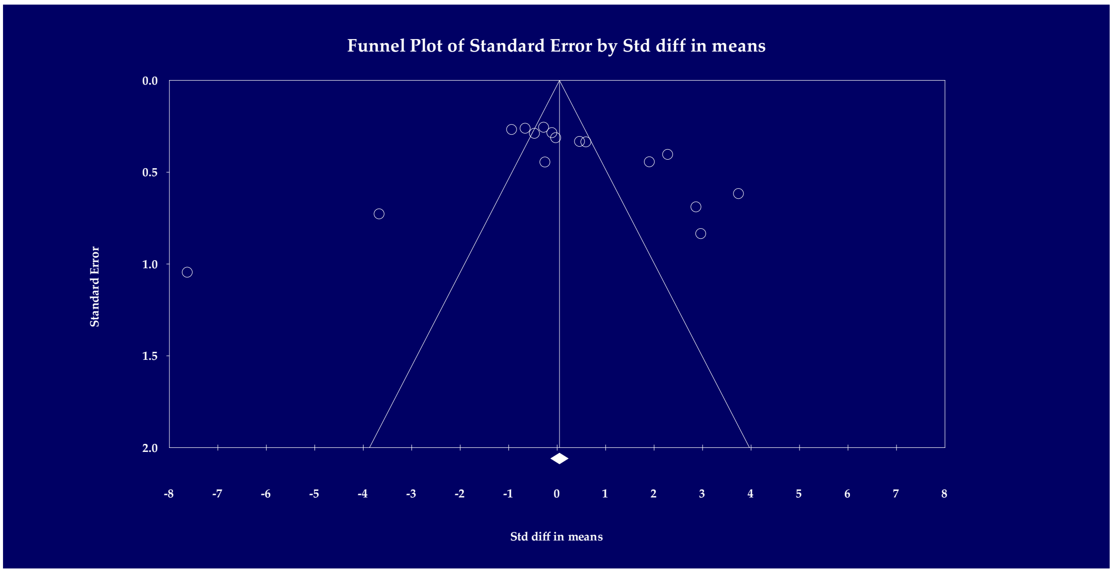

Egger's Test of the Intercept

In this case the intercept (B0) is 1.67106, 95% confidence interval (-4.26292, 7.60503), with t=0.60399, df=14. The 1-tailed p-value (recommended) is 0.27776, and the 2-tailed p-value is 0.55551.

Figure S5b: Publication bias of AST

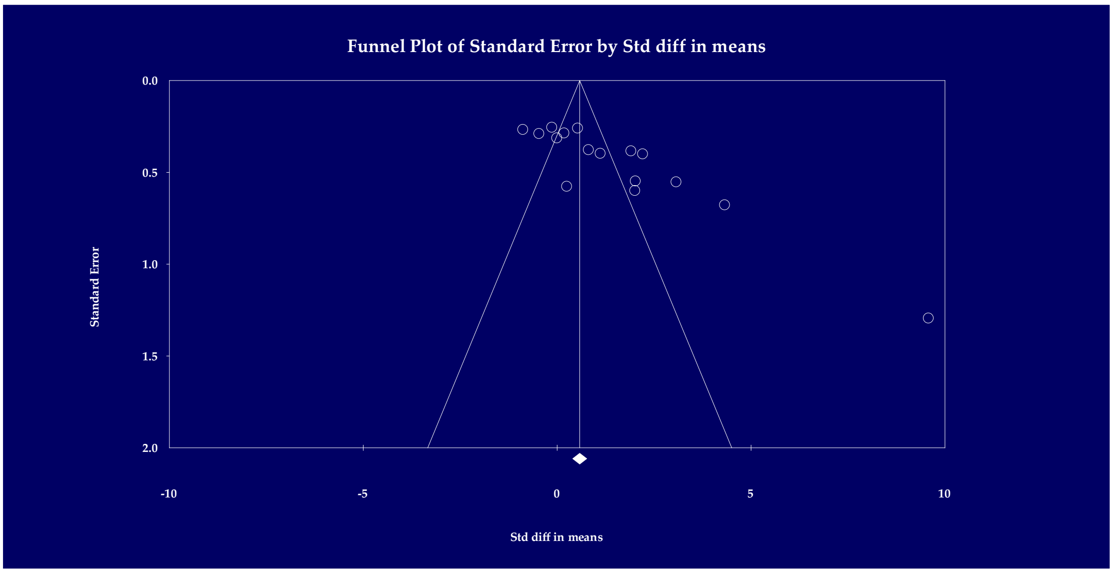

Egger's Test of the Intercept

In this case the intercept (B0) is 8.72499, 95% confidence interval (5.53714, 11.91284), with t=5.87018, df=14. The 1-tailed p-value (recommended) is 0.00002, and the 2-tailed p-value is 0.00004.

Figure S5c: Publication bias of MDA

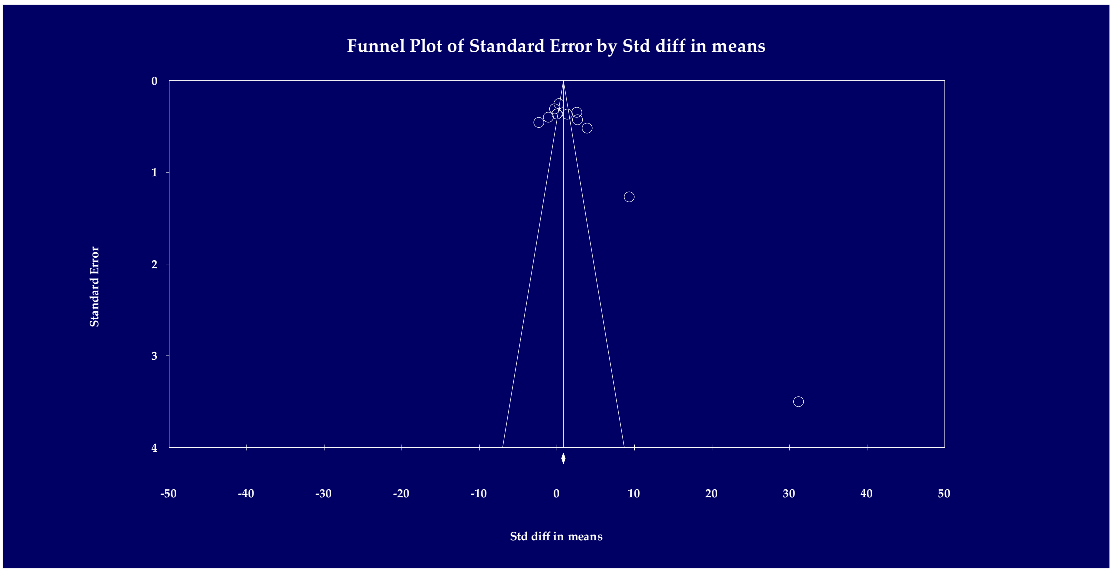

Egger's Test of the Intercept

In this case the intercept (B0) is 8.55624, 95% confidence interval (1.08999, 16.02248), with t=2.59241, df=9. The 1-tailed p-value (recommended) is 0.01455, and the 2-tailed p-value is 0.02910.

Figure S5d: Publication bias of TNF-α

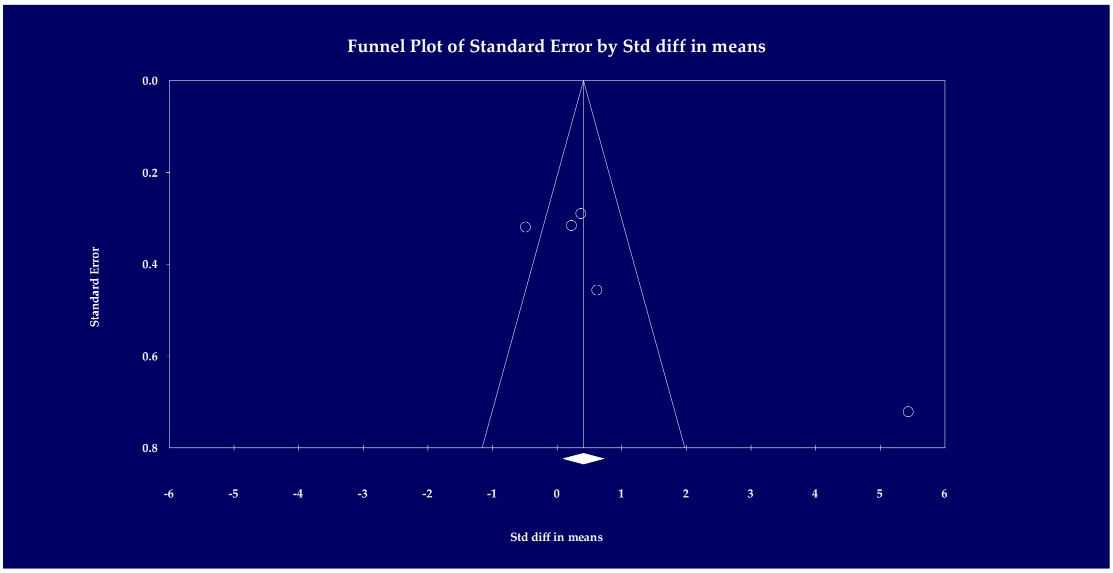

Egger's Test of the Intercept

In this case the intercept (B0) is 10.58267, 95% confidence interval (-0.55216, 21.71749), with t=3.02464, df=3. The 1-tailed p-value (recommended) is 0.02828, and the 2-tailed p-value is 0.05655.
